# Poly(ADP-ribose)polymerase 2 is zinc-dependent enzyme and nucleosome reorganizer

**DOI:** 10.1007/s00018-025-05785-8

**Published:** 2025-06-30

**Authors:** Natalya Maluchenko, Alexandra Saulina, Olga Geraskina, Elena Kotova, Anna Korovina, Grigoriy Armeev, Mikhail Kirpichnikov, Alexey Feofanov, Vasily Studitsky

**Affiliations:** 1 Faculty of Biology, Lomonosov Moscow State University, Moscow, 119234 Russia; 2 Shemyakin-Ovchinnikov Institute of Bioorganic Chemistry, Russian Academy of Sciences, Moscow, 117997 Russia; 3 Fox Chase Cancer Center, Philadelphia, PA 19111-2497 USA

**Keywords:** PARP2, Zn^2+^, Mg^2+^, AutoPARylation, SpFRET microscopy, EMSA, WGR domain

## Abstract

Poly(ADP-ribose)polymerase 2 (PARP2) is a nuclear protein, DNA damage sensor and an emerging target for development of anti-cancer drugs. Previously it was discovered that PARP2 binds to nucleosomes; however, critical factors involved in this process remain unknown. We demonstrated that in the presence of Mg^2+^ or Ca^2+^ ions PARP2 forms complexes with a nucleosome containing different number of PARP2 molecules without altering conformation of nucleosomal DNA. In contrast, Zn^2+^ ions directly interact with PARP2 inducing a local alteration of the secondary structure of the protein and PARP2-mediated, reversible structural reorganization of nucleosomes. WGR domain of PARP2 is the target for Zn^2+^ ions since this domain contains two putative Zn^2+−^binding sites, binds Zn^2+^ ions and alone drives Zn^2+^-mediated reorganization of nucleosomes. Auto(poly-ADP-ribosylation) activity of PARP2 is enhanced by Mg^2+^ ions and modulated by Zn^2+^ ions: suppressed or enhanced depending on the occupancy of two functionally different zinc binding sites. The data suggest that transient changes in concentration of cations can differentially modulate PARP2 activity, local chromatin structure and the DNA damage response.

**Graphical abstract:**

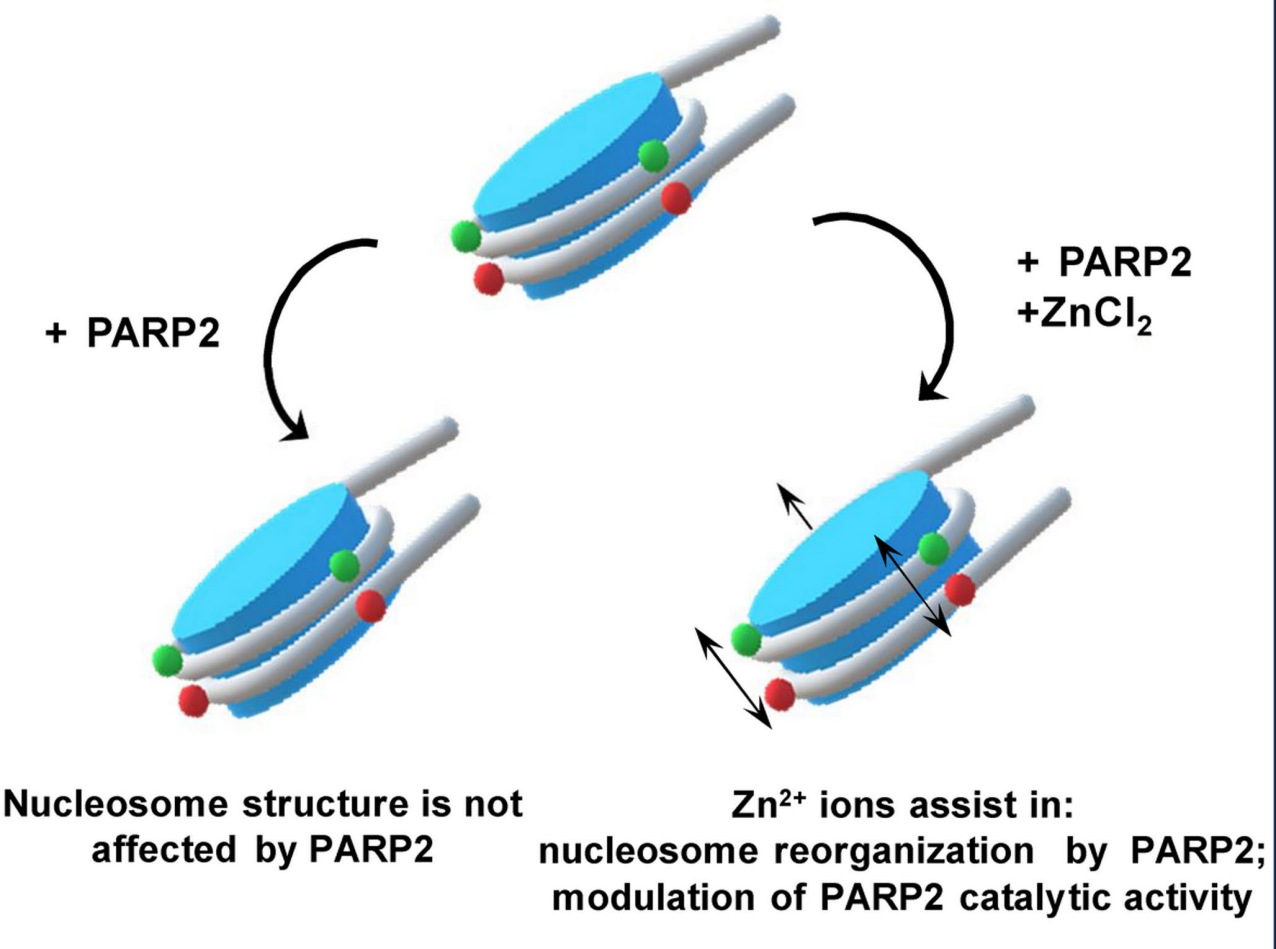

**Supplementary Information:**

The online version contains supplementary material available at 10.1007/s00018-025-05785-8.

## Introduction

PARP2 is a nuclear enzyme and a member of the poly(ADP-ribose) polymerase (PARP) family proteins. PARP2 has a multi-domain structure, which includes N-terminal region (NTR), tryptophan-glycine–arginine-rich (WGR), α-helical and catalytic domains. PARP2 plays an important role in maintaining genome stability, acting together with PARP1 as damage sensors that recruit DNA repair enzymes to the site of damage [1–4]. PARP2 catalyzes the polyADP-ribosylation (PARylation) of various proteins including PARP2 itself and histones [5] and is responsible for the synthesis of 15–25% of the poly(ADP-ribose)polymer (PAR) in cells [1, 6]. It is well known that PAR-polymers are an essential component of various cell signalling systems [7]. PARP2 is able to partially compensate for the DNA repair functions of PARP1 after knockout of PARP1, while a double knockout of PARP1 and PARP2 is lethal [8].The inhibitors that affect catalytic activity of both PARP1 and PARP2 are used in clinics for the treatment of cancers with aberrations in DNA damage repair mechanisms [9–11]. These inhibitors may produce side effects associated with the development of hematological toxicity [12–15], severe anemia [16] and a number of gastrointestinal symptoms [17]. Recent studies show that the undesirable effects can be reduced if PARP1 is inhibited selectively [18]. PARP2 is also involved in various processes including spermatogenesis [19], adipogenesis [20], thymocyte survival [21] and endometrial receptivity [22]. It triggers reactions that promote cell adaptation to stress [23].

Activation of PARP2 follows its binding to DNA. Recent studies have shown that PARP2 uses the WGR and NTR domains for DNA and nucleosome recognition [2, 24–26]. WGR plays a key role in PARP2 binding to DNA and subsequent DNA-dependent activation of PARP2 catalytic activity [26, 27]. WGR binding to DNA destabilizes the α-helical regulatory domain and allows the catalytic domain to bind the substrate (NAD+) and carry out PARylation. Since genomic DNA exists in a cell in the form of chromatin, nucleosomes are inevitably involved in PARP2-DNA interaction. Investigation demonstrate that the affinity of PARP2 for nucleosomal DNA is higher than for histone-free DNA [28]. Further studies are required to fully understand the mechanisms of interaction between PARP2 and nucleosomes as well as the factors that affect the enzyme activity.

Here we report that, despite the apparent absence of the specialized Zn^2+^-binding domains (e.g. zinc fingers), interaction of PARP2 with nucleosomes is modulated by Zn^2+^ ions. Zn^2+^ ions directly bind to PARP2 and induce subtle changes in the secondary structure of the enzyme. Furthermore, PARP2 binding to a nucleosome in the presence of Zn^2+^ ions (but not Ca^2+^ or Mg^2+^ ions) is accompanied by changes in the structure of nucleosomal DNA. Computer analysis predicts the presence of two zinc-binding sites in the WGR domain. The studies reveal Zn^2+^ binding to and the concomitant structural changes in the WGR domain, which result in the WGR-mediated reorganization of the nucleosomes. PARylation activity of PARP2 is considerably enhanced by Mg^2+^ ions. Zn^2+^ ions suppress or enhance it depending on the occupancy of two functionally different Zn^2+^ binding sites.

## Methods

### Reagents

The following reagents were used: ethylenediaminetetraacetic acid (EDTA) (Amresco, USA), ZnCl_2_ (Fluka, Switzerland), CaCl_2_, MgCl_2_, NAD+, sodium dodecyl sulfate (SDS), dithiothreitol (DTT), tris(2-carboxyethyl) phosphine (TCEP) (Sigma Aldrich, USA), mouse monoclonal antibodies against PAR (clone 10 H, Tulip BioLabs, USA), anti-mouse antibodies conjugated with horseradish peroxidase (Bio-Rad, Hercules, CA, USA).

### Proteins, DNA templates and nucleosomes

Recombinant mouse full-length PARP2 was obtained in the baculovirus expression system using Sf9 insect cells as described earlier [29] and was kindly provided by Prof. O. Lavrik. PARP2 was diluted to 2.8 µM, aliquoted in a buffer containing 40% glycerin, 32.5 mM HEPES (pH 8.0), 75 mM NaCl, 0.5 mM EDTA, 0.15 mM ZnCl_2_, 0.1 mM TCEP and stored at −80 ºC. We were cloned WGR domain of PARP2 (residues 77–220) into pET-15b-TEV expression vector containing the recognition site for TEV protease. WGR expression was induced in *Escherichia coli* Rosetta2 (DE3) pLysS strain by 0.5 mM IPTG. Lysate was applied to the HiTrap Chelating affinity chromatography column (Cytiva, USA), and the column was washed sequentially with three buffers (25 mM HEPES pH 8.0, 0.5 mM TCEP, 20 mM imidazole, 1 mM phenylmethylsulphonyl fluoride, and a protease inhibitor cocktail (Sigma P8849, USA)) containing 500, 1000 and again 500 mM NaCl. WGR was eluted with a buffer containing 25 mM HEPES pH 8.0, 500 mM NaCl, 0.5 mM TCEP, 250 mM imidazole. Then, the sample was applied to the HiTrap heparin column (Cytiva, USA) and washed with a buffer containing 50 mM Tris-HCl pH 7.0, 1 mM EDTA, 0.1 mM TCEP and the increasing gradient of NaCl (from 150 to 1000 mM). The fractions that contained WGR incubated with TEV protease and after were applied to the HiPrep 16/60 Sephacryl S-200 h gel filtration column and eluted with a gel filtration buffer (25 mM HEPES pH 8.0, 300 mM NaCl, 1 mM EDTA, 0.1 mM TCEP). The purity and homogeneity of the fractions were analyzed by SDS-PAGE. The WGR-containing fractions were concentrated, flash-frozen and stored at −80 °C.

Fluorescently labeled 187-bp DNA templates were obtained by polymerase chain reaction (PCR) using the plasmid containing nucleosome-positioning sequence s603-42 A [30] and fluorescently labeled oligonucleotides as primers. The following oligonucleotides (Lumiprobe, Russia) were used:

**Table:** 

NP_forward	5’-CAAGCGACACCGGCACTGGGCCCGGTTCGCGC[Cy3-dT]CCCGCCTTCCGTGTGTTGTCGTCTCTCGGGCGT−3’
NP_reverse	5’-GAACCATGATGGGCACTGGGTACCCCAGGGACTTGAAGTAATAAGGACGGAGGGCCTCTTTCAACATCGATGCACGG[Cy5-dT]GGTTAG−3’
Nm_forward	5’-CAAGCGACACCGGCACTGGGCCCGGTTCGCGCTCCCGCCTTCCGTGTGTTGTCG[Cy5-dT]CTCTC−3’
Nm_reverse	5’-GAACCATGATGGGCACTGGGTACCCCAGGGACTTGAAGTAATAAGGACGGAGGGCC[Cy3-dT]CTTTC−3’
Nd_forward	5’-CAAGCGACACCGGCACTGGGCCCGGTTCGCGCTCCCGCCTTCCGTGTGTTGTCGTC TCTCGGGCGTCTAAGTACGC[Cy3-dT]TAGGC−3’
Nd_reverse	5’-GAACCATGATGGGCACTGGGTACCCCAGGGACT[Cy5-dT]GAAGTAATAAGGAC−3’

PCR products were purified from 2% agarose gel and extracted by QIAquick Gel Extraction Kit (Qiagen) following the manufacturer’s protocol.

Nucleosomes were assembled using fluorescently-labeled DNA templates and chicken donor chromatin without linker histone H1 as described earlier [31]. Assembled nucleosomes were purified and stored at 4 °C.

### Single-particle Förster resonance energy transfer (spFRET) in solution

In the study of nucleosome-PARP2 interactions, nucleosomes (1–2 nM) were incubated with PARP2 (12.5–200 nM) in buffer A (50 mM Tris-HCl pH 7.5; 40 mM NaCl; 0.5 mM β-mercaptoethanol (β-ME); 0.1% NP40) at 25 °C for 30 min. When indicated, buffer A was supplemented with 50 µM – 5 mM ZnCl_2_, 5 mM CaCl_2_ or 5 mM MgCl_2_. To study the stability of Zn^2+^-PARP2-nucleosome complexes, PARP2-nucleosome complexes that have been formed for 30 min in buffer A containing 50 nM PARP2 and 0.15 mМ Zn^2+^ ions were mixed with EDTA (10 mM) and incubated for 15 min. In the studies of PARylation, NAD+ (0.5–10 µM) was mixed with nucleosomes (1–2 nM) and PARP2 (100 nM) in buffer A and incubated for 45 min at 25 °C.

spFRET measurements in solution were performed as described previously [32]. Each measured single nucleosome was characterized by FRET between Cy3 and Cy5 labels calculated as a proximity ratio (E_PR_):

1 $$\:{\text{E}}_{\text{P}\text{R}}=\left({\text{I}}_{5}-0.19\times\:{\text{I}}_{3}\right)\:/\:\left({\text{I}}_{5}+0.81\times\:{\text{I}}_{3}\right),$$

Where I_3_ and I_5_ are fluorescence intensities of Cy3 and Cy5, respectively, and coefficients 0.19 and 0.81 provide correction for the spectral cross-talk between Cy3 and Cy5 detection channels. E_PR_ is a FRET efficiency not corrected for quantum yields of labels and an instrumentation factor. Relative frequency distributions of nucleosomes by E_PR_ values, i.e. E_PR_ profiles, (2000–5000 nucleosomes per experiment; 3 independent experiments) were plotted and further analyzed as a superposition of several normal (Gaussian) distributions for nucleosomes or nucleosome-PARP2 complexes. Since nucleosomes tend to aggregate when PARP2 is added (especially at high concentrations), treatment of signals measured from nucleosomes and their complexes included mandatory rejection of signals from any aggregates that are clearly recognized due to their slow diffusion rate and/or abnormally high fluorescence intensity [33].

### EMSA (Electrophoretic Mobility Shift Assay) experiments

Sample preparation for EMSA was the same as for spFRET microscopy, although a slightly higher concentration of nucleosomes (2–3 nM) was used. The PARP2-nucleosome complexes were subjected to 5% polyacrylamide gel in 0.2×TBE buffer. Electrophoresis was performed under native conditions at 140 V, + 4 °C for ∼1.5 h. The gels were scanned using Amersham Typhoon RGB imager (Cytiva, Sweden). Fluorescence was excited in the gel at the 532 nm wavelength and recorded in the 570–610 nm (Cy3 signal) and 650–700 nm (FRET signal of Cy5) spectral regions. The resulting images were obtained in green (Cy3 signal) and red (FRET signal of Cy5) colors and merged.

### Single particle fluorescence intensity analysis of nucleosomes in the gel

For the analysis of stoichiometry of the PARP2-nucleosome complexes, gels obtained in EMSA experiments were placed between object and cover glasses and subjected to single particle fluorescence intensity analysis as described previously [34]. Measurements were performed using the 633 nm excitation wavelength and the 650–800 nm detection range, thus exciting and detecting fluorescence intensity of Cy5 (I_Cy5_) only. The measured sets of data were presented as relative frequency distributions of the complexes by I_Cy5_, i.e. I_Cy5_ profiles. This approach made it possible to estimate the number of nucleosomes per single particle (PARP2-nucleosome complex) for each gel band and make conclusions about the stoichiometry of PARP2-nucleosome complexes separated in gel.

### Western blots (WB)

Nucleosomes (3 nM) were preincubated with PARP2 (100 nM) in the buffer A supplemented with 5 mM MgCl_2_ or 0.3 mM ZnCl_2_ when indicated for 30 min followed by 5 min incubation with 10 mM EDTA (when indicated) and 30 min incubation with NAD+ (0, 1, 2.5 or 5 µM) in the same buffer. Probes were subjected to electrophoresis in 4–12% bis-Tris gradient gel in the NuPAGE™ MES SDS Running Buffer at 130 V. Protein transfer on polyvinylidene fluoride membrane was performed in the transfer buffer (25 mM Bicine, 25 mM Bis-Tris (free base), 1 mM EDTA pH 7.2) containing 20% methanol at 4 °C and 70 V for 1 h. The membrane was incubated for 60 min in the PBS-T solution supplemented with the 5% skimmed milk. Then the membrane was incubated with mouse monoclonal antibodies 10 H followed by incubation with the secondary anti-mouse antibodies conjugated with horseradish peroxidase (Bio-Rad, Hercules, CA, USA). The washing procedure was carried out after each step of incubation. Immunodetection was performed using the Super Signal West Pico Chemiluminescent Substrate (Thermo Fisher Scientific, Waltham, MA, USA) for 3 min. The Western blot experiments were performed in three independent repetitions.

### Circular dichroism (CD) spectroscopy

Samples of PARP2 (5 µM) were prepared in a buffer containing 5 mM HEPES pH 7.5, 30 mM NaCl, 0.2 mM EDTA, 0.02 mM TCEP and (when indicated) 0.7 mM ZnCl_2_, CaCl_2_ or MgCl_2_. Samples of WGR domain (45 µM) were prepared in a buffer containing 18.7 mM Tris pH 8.0, 1.6 mM HEPES, 103 mM NaCl, 70 µM EDTA, 7 µM TCEP and (when indicated) 5 mM CaCl_2_ or 5 mM MgCl_2_ or 0.3 mM ZnCl_2_.

CD spectra of proteins were recorded within the 190–250 nm spectral range with 0.2 nm step using Jasco-810 spectrophotometer (Jasco, Japan) and the quartz SUPRASIL^®^ cuvette of 0.1 mm thickness (Hellma, Germany.

Secondary structure analysis was performed using BeStSel webserver [35]. To improve the reliability of the analysis, the content of canonical secondary structures of various types was predicted for 3–6 independent measurements, and the values were averaged. The statistical significance of the secondary structure difference was determined using the multiple t-test.

### Fluorescence spectroscopy

Fluorescence spectra of Trp residues were measured in the 300–450 nm spectral range at the 270 nm excitation wavelengths using the Cary Eclipse spectrofluorometer (Varian/Agilent, USA).

Fluorescence of PARP2 was measured at 0.5 µM in a buffer containing 7% glycerin, 33 µM HEPES (pH 8.0), 75 mM NaCl, 89 µM EDTA, 27 µM ZnCl_2_, 1.5 µM TCEP. A solution of PARP2 was complemented with ZnCl_2_ to 377 µM.

Fluorescence of WGR domain was measured at 0.5 or 5 µM in a buffer containing 10 mM HEPES (pH 8.0), 150 mM NaCl and 0.73 or 7.3 µM EDTA, respectively. Solutions were complemented with ZnCl_2_ (0.2–308 µM) or MgCl_2_ (15–5000 µM), or CaCl_2_ (8–5000 µM) dissolved in a buffer containing 10 mM HEPES (pH 8.0), 150 mM NaCl. Intensity of the measured spectra was corrected for the concomitant dilution of the WGR domain. Integral intensities (*I*_*fl.*_) of fluorescence spectra were averaged over three independent measurements (mean ± SEM) and used to plot the corresponding *I*_*fl.*_ vs. ion concentration graphs. Data were fitted with the one site binding equation:

2 $$\:{I}_{fl}\left(\text{C}\right)={I}_{m}C/\left({K}_{d}+C\right)+{I}_{fl0},$$

Where *C* is the concentration of divalent cations, *K*_*d*_ is a dissociation constant of the complex, *I*_*m*_ and *I*_*fl0*_ are maximal and initial values of *I*_*fl.*_, respectively.

### Molecular modeling of zinc-binding sites in the WGR domain of PARP2

The initial model of the WGR domain bound to zinc ions in putative binding sites was constructed on the basis of the protein data base (PDB) structure 6F5B using ChimeraX 1.6.1 [36] with the ISOLDE 1.6.0 extension [37]. To achieve this, all chains except chain A were removed from the file with the 6F5B structure. In putative binding site 1, a zinc atom was placed at the geometric centre between the OE1 atom of residue E97, the SG atom of residue C98, and the NE2 atom of residue H160. Similarly, a zinc atom was positioned between residues H106, C109, and E138 in putative binding site 2. Constraints with a target distance of 3 Å and a stiffness constant of 50 kJ mol^−1^ nm^−1^ were added between the listed atoms and the zinc atom. Molecular dynamics (MD) simulations were initiated using standard ISOLDE potentials for the environment within ± 10 amino acids of the putative zinc binding sites. To model the free WGR domain, chain A of the 6F5B model was used without additional modifications.

The resulting model was prepared for MD simulations using GROMACS 2022.1 software [38] with the Amber14sb force field [39] and additional corrections for the zinc binding sites proposed earlier [40, 41]. The TIP3P water model was employed [42]. Both systems were placed in truncated octahedral simulation boxes with periodic boundary conditions set at least 2 nm away from the WGR domain atoms. Water molecules were added for solvation, and Na^+^ and Cl^−^ions were introduced to neutralize the charge and achieve ionic strength of 150 mM. Minimization was conducted using the Broyden–Fletcher–Goldfarb–Shanno (BFGS) method for 10,000 steps. Subsequently, the system underwent equilibration in five steps with gradual reduction of positional restraints: 100 ps with restraints on heavy atoms at 500 kJ mol^−1^ nm^−2^ with the 0.5 fs timestep; 200 ps with restraints at 50 kJ mol^−1^ nm^−2^ with the 2 fs timestep (and continued in the same manner); 200 ps with restraints at 5 kJ mol^−1^ nm^−2^; 200 ps with restraints at 0.5 kJ mol^−1^ nm^−2^; 200 ps of unrestrained simulations. Temperature was maintained at 300 K using the velocity rescale scheme, and pressure at 1 bar using the Parrinello-Rahman barostat. The Particle Mesh Ewald method was used for Coulomb interactions (0.8 nm real space cut-off, fourth degree PME order, 0.12 nm Fourier spacing). Bond constraints were applied using the fourth-order LINear Constraint Solver algorithm with one iteration. MD production runs employed a 2 fs integration timestep, and trajectory frames were saved every 1 ns for 400 ns. Root-mean-square deviation (RMSD) values and radius of gyration were calculated using Python scripts with the MD analysis library. The initial 100 ns were omitted from the latter analysis to exclude possible non-equilibrium conformations. Solvent accessible surface areas were determined using a Python script based on the Free SASA 2.1.0 library [43]. Secondary structure composition was calculated using the DSSP 3.1.5 [44] program for each saved frame. Visualization was performed in ChimeraX 1.6.1 software.

## Results

### Experimental approaches

**Fig. 1:**
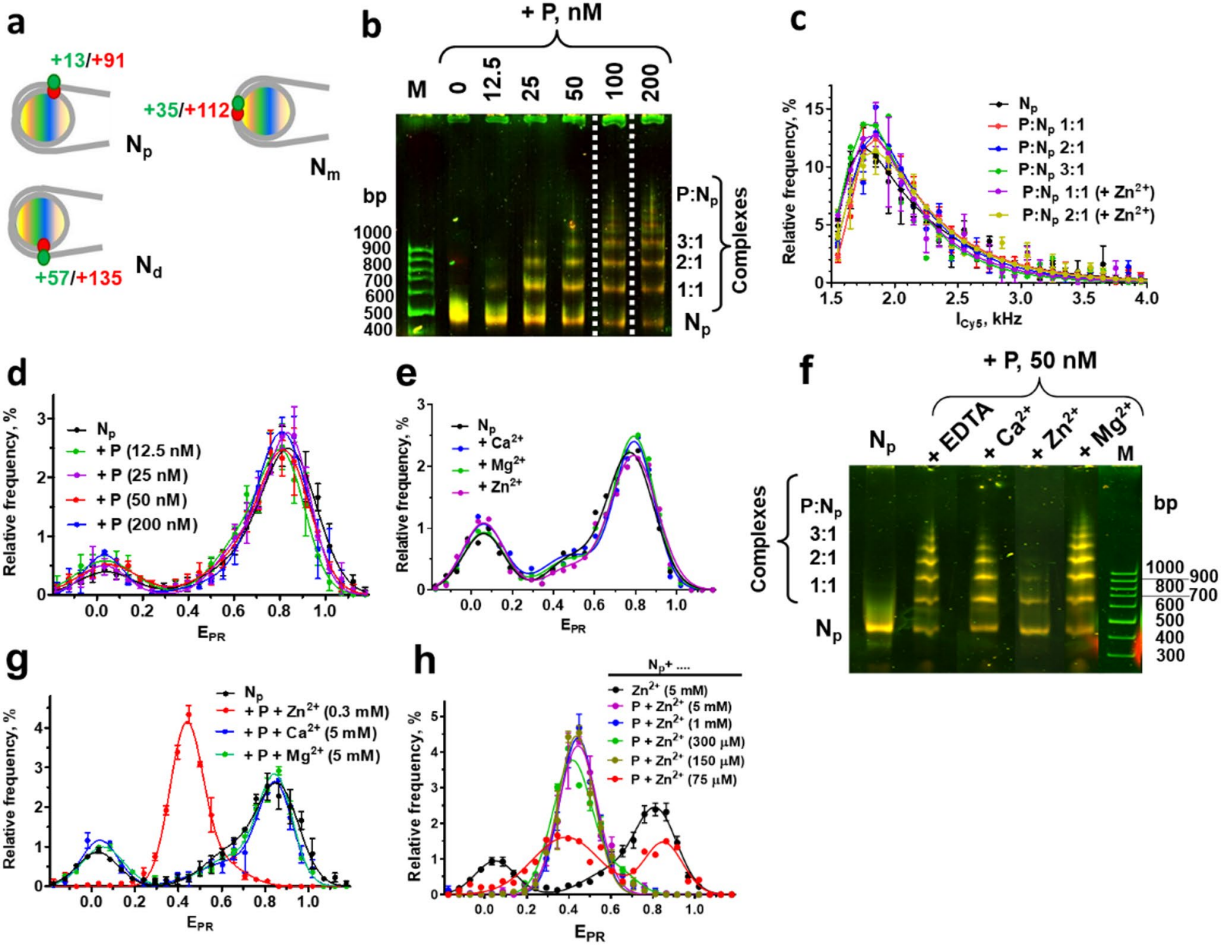
Multiple PARP2 molecules can bind to a nucleosome and induce Zn^2+^-dependent nucleosome reorganization. **(a)** Positions of Cy3 (green) and Cy5 (red) labels in the nucleosomes. Numbers indicate distances (bp) from the boundary of nucleosomal DNA to the labeled nucleotides. **(b)** Analysis of N_P_ nucleosomes and their complexes with PARP2 (P, 12.5–200 nM) formed in the absence of divalent ions by non-denaturing PAGE. The proposed stoichiometry of the PARP2-nucleosome complexes (P: N_P_) in the gel is indicated. M – DNA markers. **(c)** Frequency distributions of N_P_ nucleosomes and their complexes with PARP2 by the Cy5 fluorescence intensity (I_Cy5_) that were measured by single particle fluorescence microscopy within different bands of the gels shown in Fig. 1b, f. **(d)** E_PR_ profiles of N_P_ nucleosomes measured with spFRET microscopy in the absence or presence of PARP2 (P). The concentration of PARP2 varied from 12.5 to 200 nM. **(e)** E_PR_ profiles of N_P_ nucleosomes in the absence of divalent ions or in the presence of 5 mM Zn^2+^, Ca^2+^ or Mg^2+^ ions. **(f)** Analysis of N_P_ nucleosomes and their complexes with PARP2 (P, 50 nM) formed in the absence of divalent ions (N_p_) or in the presence of Zn^2+^ (0.3 mM), Ca^2+^ (5 mM) or Mg^2+^ (5 mM) ions by non-denaturing PAGE. The proposed stoichiometry of the PARP2-nucleosome complexes (P: N_P_) in the gel is indicated. M – DNA markers. **g)** E_PR_ profiles of N_P_ nucleosomes and their complexes with PARP2 (P, 50 nM) in the presence of Zn^2+^ (0.3 mM), Ca^2+^ (5 mM) or Mg^2+^ (5 mM). **h)** E_PR_ profiles of N_P_ nucleosomes and their complexes with PARP2 (P, 50 nM) in the absence or presence of different concentrations of Zn^2+^ions. **(c-e**,** g**,** h)** Statistics: mean ± SEM; 3 independent experiments; 2000–5000 particles per experiment

For spFRET and EMSA the nucleosomes were labeled with a donor-acceptor pair of Cy3 and Cy5 fluorophores (Fig. 1a) attached to the neighboring gyres of nucleosomal DNA at the following positions: 13 and 91 bp (N_p_ nucleosomes), 35 and 112 bp (N_m_ nucleosomes) or 57 and 135 bp (N_d_ nucleosomes) from the boundary of the 603 nucleosome positioning DNA sequence [7, 32]. The labels placed at these positions support efficient FRET and allow analysis of structural changes near and far from the boundary of the nucleosomal DNA [7, 32, 45]. Single nucleosomes or their complexes with PARP2 freely diffusing in a solution were subjected to spFRET measurements and the data are presented as E_PR_ profiles (Fig. 1d, g).

### Multiple PARP2 molecules can bind to a nucleosome in the absence of divalent ions

PARP2 forms complexes with nucleosomes at the concentration of the enzyme of ~ 12.5 nM and higher in the absence of divalent ions (Fig. 1b). A ~ 50% decrease in the number of free nucleosomes is observed at 40 ± 10 nM PARP2. An increase in PARP2 concentration is accompanied by formation of multiple complexes having different electrophoretic mobilities in the gel (Fig. 1b). To track alterations in the structure of nucleosomes within the complexes, we used the FRET-in-gel analysis and monitored the color of the bands in gel: it can vary from green (no FRET) to yellow (higher FRET) and to red (nearly 100% FRET), depending on the structure of the labeled nucleosomes. The bands in the gel corresponding to free nucleosomes and different PARP2-nucleosome complexes have similar yellow color, suggesting that different complexes have similar structures of nucleosomal DNA (Fig. 1b). Since formation of multiple complexes can indicate different stoichiometry of interacting components, we have analyzed fluorescence intensity of single complexes (I_Cy5_) within bands in gel to evaluate whether the number of nucleosomes in the complexes with PARP2 varies. The fluorescence of Cy5 label was selectively excited and measured with single particle fluorescence microscopy. Comparison of I_Cy5_ profiles of the complexes within different bands corresponding to various complexes did not reveal significant differences (Fig. 1c). The data suggest that different complexes observed in the gel contain one nucleosome per complex, and the different mobilities are explained by binding of different number of PARP2 molecules to the nucleosome (Fig. 1b, c).

Consistently, the spFRET data indicate absence of structural changes in nucleosomes upon formation of the complexes with PARP2 in the absence of divalent ions: the E_PR_ profiles of nucleosomes are very similar in the absence and presence of PARP2 and reveal two subpopulations of nucleosomes in the solution (Fig. 1d). Major subpopulation is characterized by higher E_PR_ values with maximum at 0.8 and corresponds to nucleosomes having neighboring gyres of nucleosomal DNA near each other at the labeled sites. Minor subpopulation of the nucleosomes is characterized by significantly lower E_PR_ values with a maximum at 0.03 and corresponds to histone-free DNA and/or nucleosomes, in which the distance between DNA gyres increased considerably, likely due to so-called nucleosome breathing [46]. SpFRET analysis shows absence of structural changes in nucleosomes in the wide range of PARP2 concentrations (12.5–200 nM, Fig. 1d), when various types of complexes of different stoichiometry are formed according to EMSA data (Fig. 1b).

Taken together, the data suggest that in the absence of divalent cations several PARP2 molecules bind to a nucleosome without affecting the conformation of nucleosomal DNA.

### Zn^2+^ selectively affects the structure of the nucleosome-PARP2 complexes

The nucleoplasm contains submillimolar concentrations of Ca^2+^, Mg^2+^ and Zn^2+^ cations that could modulate the structure of chromatin and the activity of nuclear proteins [47–50]. Millimolar concentrations of Ca^2+^, Mg^2+^ or Zn^2+^ ions minimally affect the nucleosome structure (Fig. 1e). Formation of multiple PARP2-nucleosome complexes with different stoichiometry is observed in the presence of Ca^2+^ or Mg^2+^ ions (Fig. 1f); the structure of N_P_ nucleosomes is not changed in these complexes (Fig. 1f, g). In contrast, Zn^2+^ ions induce alterations in the structure of PARP2-nucleosome complexes (Fig. 1g, h). Two types of PARP2-nucleosome complexes are formed in the presence of Zn^2+^ ions (Fig. 1f). Both complexes contain one nucleosome (Fig. 1c) and, therefore, differ in the number of PARP2 molecules per nucleosome (1:1 and 2:1). Probably, complexes with a higher molar ratio of PARP2 to nucleosome are formed in the presence of Zn^2+^; however, aggregation at a concentration of PARP2 above 50 nM prevents their separation in the gel (Figure S1). The changes in the nucleosome structure induced by combination of PARP2 and Zn^2+^ result in the appearance of a new peak centered at E_PR_=0.4 in the E_PR_ profile (Fig. 1g, h). This peak corresponds to a subpopulation of N_P_ nucleosomes that have significantly increased distance between DNA gyres near the location of fluorescent labels.

The data show that Zn^2+^ ions, but not Ca^2+^ and Mg^2+^ ions, specifically affect the interaction of PARP2 with nucleosomes.

### Interactions of PARP2 with nucleosomes in the presence of Zn^2+^ ions

The unusual effect of Zn^2+^ ions on nucleosome structure was further studied. By decreasing the concentration of Zn^2+^ ions in the solution, we found that Zn^2+^ ions affect the structure of PARP2-nucleosome complexes, when the concentration of Zn^2+^ is higher than 75 µM (Fig. 1h). At a concentration of 150 µM, all the complexes are converted to the altered conformation. An increase in the concentration of Zn^2+^ up to 5 mM did not cause any additional changes in the structure of the complexes (Fig. 1h).

Dilution of PARP2 to 50 nM, together with Zn^2+^ ions (0.15 mM), TCEP (0.1 mM) and EDTA (0.5 mM), which are all present in the concentrated protein sample, results in a background level of Zn^2+^ (2.7 µM), TCEP (1.8 µM) and EDTA (9 µM) in our reaction mixtures (Fig. 1g, h). The background Zn^2+^ ions are chelated with EDTA and therefore cannot affect the results of our measurements. The remaining amount of Zn^2+^ chelating agents (EDTA and TCEP) affects experiments at micromolar concentrations of added Zn^2+^ ions. It is likely that this factor limits the detection of structural reorganization in the PARP2-nucleosome complex when the concentration of added Zn^2+^ is below 75 µM.

**Fig. 2:**
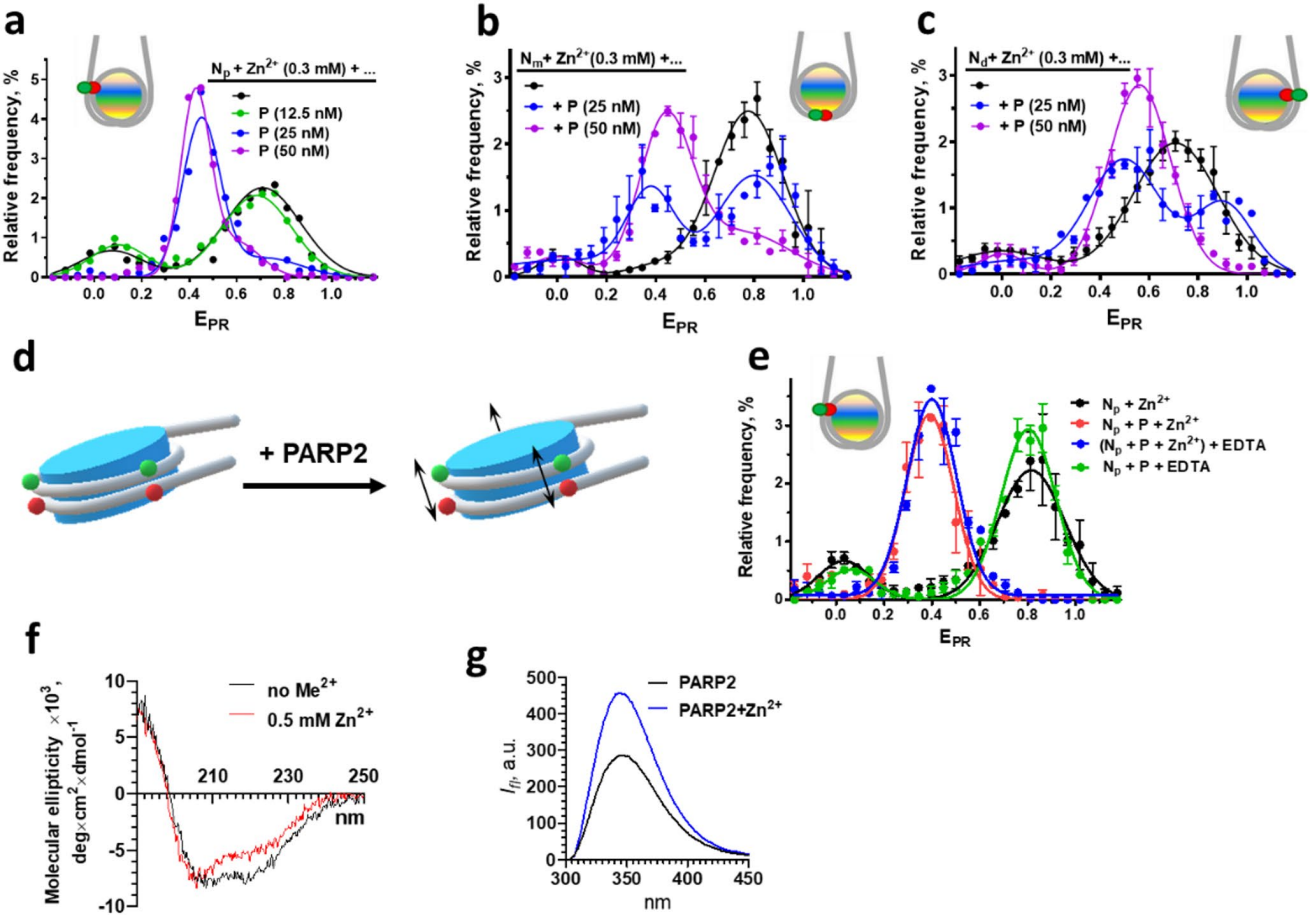
Binding of Zn^2+^ affects structures of PARP2 and PARP2-nucleosome complex. **a-c)** E_PR_ profiles of N_P_ (**a**), N_m_ (**b**) and N_d_ (**c**) nucleosomes and their complexes with PARP2 (P) in the presence of 0.3 mM Zn^2+^ ions. **d)** Proposed conformational changes in nucleosomal DNA induced by PARP2 binding. Green and red circles mark positions of the labels used to detect the conformational changes. Black arrows indicate an increase in the distance between DNA gyres detected using spFRET microscopy. **e)** Dependence of E_PR_ profiles of nucleosome N_P_ complexes with PARP2 (P, 50 nM) on the presence of EDTA. Samples were measured in the presence or absence of 0.15 mM Zn^2+^ ions. EDTA (10 mM) was added to the preformed PARP2-nucleosome complexes. **f)** Changes in the CD spectrum of PARP2 (8 µM) after addition of Zn^2+^ ions (0.5 mM). Me^2+^ - any divalent cations. **g)** Zn^2+^-induced change in the fluorescence spectrum of Trp residues of PARP2. Concentrations of PARP2 and Zn^2+^ ions were 0.5 and 377 µM in a buffer containing 7% glycerin, 33 µM HEPES (pH 8.0), 75 mM NaCl, 89 µM EDTA, 1.5 µM TCEP. **a-c**,** e)** Statistics: mean ± SEM; 3 independent experiments; ~3000 particles per experiment

To characterize Zn^2+^-dependent structural changes induced by PARP2 within different regions of nucleosomal DNA (the 35/112 and 57/135 bp regions), the N_m_ and N_d_ nucleosomes (Fig. 1a) were studied (Fig. 2b, c). As in the case of the N_P_ nucleosomes (Fig. 2a), spFRET microscopy detects two subpopulations of particles for N_m_ and N_d_ nucleosomes in the presence of Zn^2+^ ions: a major subpopulation having higher E_PR_ values and a minor subpopulation having lower E_PR_ values (Fig. 2b, c). Addition of PARP2 to nucleosomes in the presence of Zn^2+^ ions resulted in formation of a rather uniform structural state of nucleosomes having E_PR_ values centered at 0.4 and 0.5 for N_m_ and N_d_ nucleosomes, respectively (Fig. 2b, c). These changes in the E_PR_ profiles of nucleosomes correspond to an increase in the distance between base pairs 35 and 112 as well as between base pairs 57 and 135 in the neighboring DNA gyres after formation of the complexes. Together with the data for N_P_ nucleosomes (Figs. 1h and 2a), the results show that the structural changes induced by PARP2 in the presence of Zn^2+^ ions affect the entire nucleosomal DNA (Fig. 2d).

In the presence of Zn^2+^ ions, the formation of complexes between PARP2 and nucleosomes occurs at PARP2 concentration in the range from 12.5 to 50 nM (Fig. 2a-c). Since similar concentration dependence of PARP2 binding to nucleosomes was observed in the absence of Zn^2+^ (Fig. 1b), the affinity of PARP2 to nucleosomes is not significantly affected by Zn^2+^ ions. At the same time, Zn^2+^ ions induce a significant change in the structure of nucleosomes within the PARP2-nucleosome complexes.

At least two types of PARP2-nucleosome complexes are formed in the presence of Zn^2+^ ions (Figs. 1f and S1); the conformation of nucleosomal DNA in these complexes is similar according to spFRET analysis (Fig. 2a).

EDTA has a very high affinity to Zn^2+^ ions (*K*_*d*_∼10^−16^ M) [51, 52] and competitively removes Zn^2+^ from complexes with many proteins in a few minutes after their exposure to the chelator [52, 53]. Accordingly, the addition of PARP2 to nucleosomes in the presence of both EDTA (0.15 mM) and Zn^2+^ ions (0.15 mM) has no effect on the structure of nucleosomes (data not shown). In contrast, the addition of EDTA to the PARP2-nucleosome complexes preformed in the presence of Zn^2+^ does not reverse the changes in the nucleosome structure even at the thirtyfold molar excess of EDTA over Zn^2+^ ions in solution (Fig. 2e). These results suggest that Zn^2+^ ions involved in the formation of the PARP2-nucleosome complex are hidden inside the complex and therefore are not accessible to EDTA.

Since the structure of nucleosomes is not affected in the presence of up to 5 mM Zn^2+^ ions (Fig. 1e), and the structural changes induced in nucleosomes by PARP2 are essentially the same in the wide range of Zn^2+^ concentrations (75 µM – 5 mM, Fig. 1h), the binding of Zn^2+^ ions to nucleosomal DNA is unlikely to play a role in maintaining the altered structure of the nucleosome-PARP2 complex.

In summary, Zn^2+^ (but not Ca^2+^ and Mg^2+^) ions mediate the PARP2-induced structural rearrangement of nucleosomal DNA without affecting the affinity of the enzyme to nucleosomes.

### Zinc ions bind to PARP2 and affect its structure

No apparent Zn^2+^-binding domains were described for PARP2. At the same time Zn^2+^-dependent structural changes in a nucleosome induced by PARP2 binding (Fig. 2a-c) might indicate that Zn^2+^ ions specifically interact with PARP2, possibly altering conformation of the domain(s) of PARP2 that are responsible for the interaction with a nucleosome. To evaluate this possibility, circular dichroism (CD) spectra of PARP2 were measured before and after addition of 0.3 mM Zn^2+^, 0.7 mM Ca^2+^ or 0.7 mM Mg^2+^ ions. The CD spectra are similar except for the spectrum recorded in the presence of Zn^2+^ ions (Fig. 2f and S2b). In the latter case the spectral differences indicate that Zn^2+^ ions induce small conformational changes in PARP2 (Figure S2a). Direct interaction of Zn^2+^ ions with PARP2 was confirmed using fluorescence spectroscopy. Intensity of a fluorescence spectrum of tryptophan residues of PARP2 was increased after addition of Zn^2+^ ions, indicating changes in the microenvironment and/or in the interactions of tryptophan residues (Fig. 2g).Since the presence of Zn^2+^ does not affect the structure of intact nucleosomes (Fig. 1e), the Zn^2+^-induced alterations in the conformation of PARP2 likely drive the changes in the mode of PARP2 interaction with a nucleosome and reorganization of the nucleosome structure in the complex.

### WGR domain of PARP2 binds zinc ions and reorganizes nucleosome structure

**Fig. 3:**
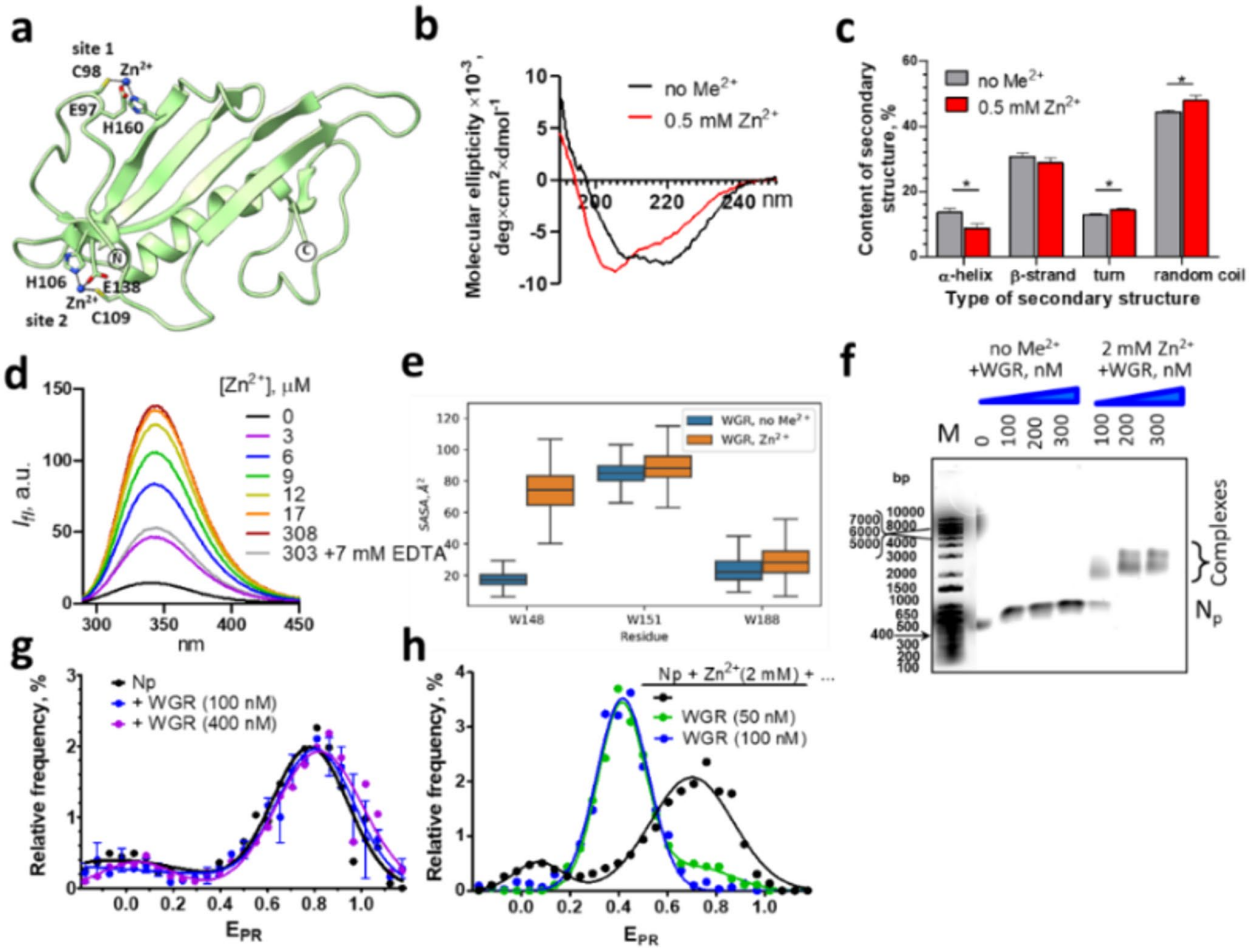
WGR domain of PARP2 binds zinc ions and reorganizes nucleosome structure. **(a)** A model of Zn^2+^ binding to the WGR domain created on the basis of the PDB structure 6F5B. Two putative binding sites are shown. N- and C-terminal ends of the WGR domain are indicated. The numbering of the residues corresponds to the numbering of the complete PARP2 sequence. **(b)** CD spectra of the WGR domain (60 µM) in a buffer containing no divalent cations (Me^2+^) or 0.5 mM Zn^2+^ ions. **(c)** Content of canonical types of secondary structures in the WGR domain of PARP2 according to the analysis of CD spectra of the WGR domain measured in the absence or presence of 0.5 mM Zn^2+^ ions. **(d)** Changes in the fluorescence spectra of tryptophan residues 148, 151 and 188 of the WGR domain (5 µM) at different concentrations of Zn^2+^ ions. **(e)** The distribution of the solvent accessible surface area (SASA) for the tryptophan residues of the WGR domain and its complex with Zn^2+^ ions during MD simulations. **(f)** Analysis of N_P_ nucleosomes and their complexes with the WGR domain in the absence of divalent cations (Me^2+^) or in the presence of 2 mM Zn^2+^ ions by electrophoresis in the 0.7% agarose gel. M – DNA markers. **(g**,** h)** Typical E_PR_ profiles of N_p_ nucleosomes and their complexes with the WGR domain formed in the absence of divalent cations (**g**) or in the presence of 2 mM Zn^2+^ ions (**h**). Statistics: ~3000 particles per experiment

Analysis of the primary PARP2 sequence using Zinc Bind Predict [54] did not reveal any Zn^2+^-binding sites (ZnBSs). However, many ZnBSs cannot be recognized from the primary sequence because they are formed only within the folded protein due to spatial proximity of zinc-coordinating amino acid residues. Zinc ions can be coordinated to histidine, cysteine, glutamate and aspartate residues [55]. Structural analysis of the WGR (PDB entry: 6F5B), catalytic and α-helical (PDB entry 4ZZY) domains of PARP2 revealed two sites potentially capable of binding zinc ions: one formed by residues E97, C98, H160 (site 1) and another - by residues H106, C109, E138 (site 2); both sites are localized in the WGR domain (Fig. 3a). However, in the WGR crystal structure, which was obtained in the absence of zinc ions [25], these residues are too far apart from each other for efficient Zn^2+^ coordination. Using a guided molecular dynamics approach, a model of the WGR domain with two Zn^2+^ ions bound to the sites 1 and 2 was developed (Fig. 3a). To assess the stability of the proposed structures, the 400 ns molecular dynamics simulations were performed for free WGR domain and WGR-Zn^2+^ complex. Both structures-maintained stability (Figure S3a) and size (Figure S3b) during the simulation time.

The proposed ZnBSs formed in the presence of Zn^2+^ can be assigned to the C1E1H1 family of ZnBSs according to the classification of a ZincBind database [56].

To evaluate the prediction about localization of ZnBS in the WGR domain, a recombinant WGR domain was purified, and its interaction with Zn^2+^ ions was studied using CD and fluorescence spectroscopy. The structure of WGR domain significantly changes in the presence of Zn^2+^ ions (Fig. 3b, c). In contrast, only minimal changes in the structure of the WGR domain were detected in the presence of Ca^2+^ or Mg^2+^ ions (Figure S2c).

Since the WGR domain contains three Trp residues, the effect of Zn^2+^ ions on intrinsic fluorescence of Trp residues was studied using fluorescence spectroscopy. Intensity of tryptophan fluorescence of the WGR domain was considerably increased after addition of Zn^2+^ ions without concomitant alterations in the shape and maximum of the fluorescence spectrum (Fig. 3d). These changes of fluorescence are similar to those observed for PARP2 (Fig. 2g), confirming the proposal about interaction of Zn^2+^ ions with the WGR domain; the interaction is accompanied by the changes in the microenvironment/interactions of one or several Trp residues. The changes in the fluorescence of Trp residues of the WGR domain were observed at the 0.4–5 µM Zn^2+^ ions (Figure S4) and the dissociation constant for Zn^2+^-WGR domain complex is 2.0 ± 0.4 µM.

The data on the changes in fluorescence of Trp residues of the WGR domain are consistent with the results of bioinformatic analysis predicting the presence of ZnBSs in the WGR domain. Furthermore, data of molecular modeling predict that the binding of Zn^2+^ ions to the WGR domain would lead to a change in the environment and to a significant increase in the accessibility of at least one tryptophan residue (Trp 148) to solvent (Fig. 3e).

Ca^2+^ and Mg^2+^ ions also bind to the WGR domain, but the effects of these ions on the intensity of Trp fluorescence is considerably lower than the effect of Zn^2+^ ions (Figure S5). Probably, sites of binding of Ca^2+^ and Mg^2+^ ion differ from the binding site(s) of Zn^2+^, and/or their binding induces smaller structural changes near Trp residues. The dissociation constants of Ca^2+^ and Mg^2+^ ion for the complexes with the WGR domain are 29 ± 14 and 180 ± 80 µM, respectively.

The WGR domain binds to nucleosomes in the presence and absence of Zn^2+^ ions: in the presence of Zn^2+^, mobility of complexes in gel decreases considerably as compared to free nucleosomes; without divalent cations, the mobilities of the complexes and nucleosomes are only slightly different (Fig. 3f). The binding of nucleosomes to the WGR domain was confirmed after immobilization of the protein containing His-tag on the surface of Ni-NTA beads using fluorescence microscopy (Figure S6). Although N_d_ nucleosomes containing fluorescent labels slightly bind to the beads in the absence of the WGR domain, the binding is strongly increased in the presence of WGR (Figure S6).

SpFRET microscopy shows that the binding of the WGR domain to nucleosomes in the absence of Zn^2+^ is not accompanied by significant changes of the nucleosome structure (Fig. 3g), while in the presence of Zn^2+^ the structure of the nucleosome in the complex with the WGR domain changes considerably (Fig. 3h). The appearance of a new peak in the E_PR_ profile with a maximum at 0.4 (Fig. 3h) suggests an increase in the distance between gyres of nucleosomal DNA. The similarity of the Zn^2+^-dependent changes in the E_PR_ profiles of PARP2-nucleosome and WGR-nucleosome complexes (Figs. 2a and 3h, respectively) suggests that the WGR domain of PARP2 is mainly responsible for the Zn^2+^-dependent reorganization of nucleosomes by PARP2.

Thus, Zn^2+^ binding sites that are responsible for the Zn^2+^-dependent changes in the conformation of PARP2 and for the reorganization of nucleosomes by PARP2 are localized in the WGR domain. The WGR domain can also bind Ca^2+^ and Mg^2+^ ions, but their affinities are lower, and the binding is not accompanied by the structural reorganization of the WGR domain, PARP2 and the PARP2-nucleosome complexes (except for the changes in the ternary structure near Trp residues in the WGR domain).

### Enzymatic activity of PARP2 in the complexes with nucleosomes

**Fig. 4:**
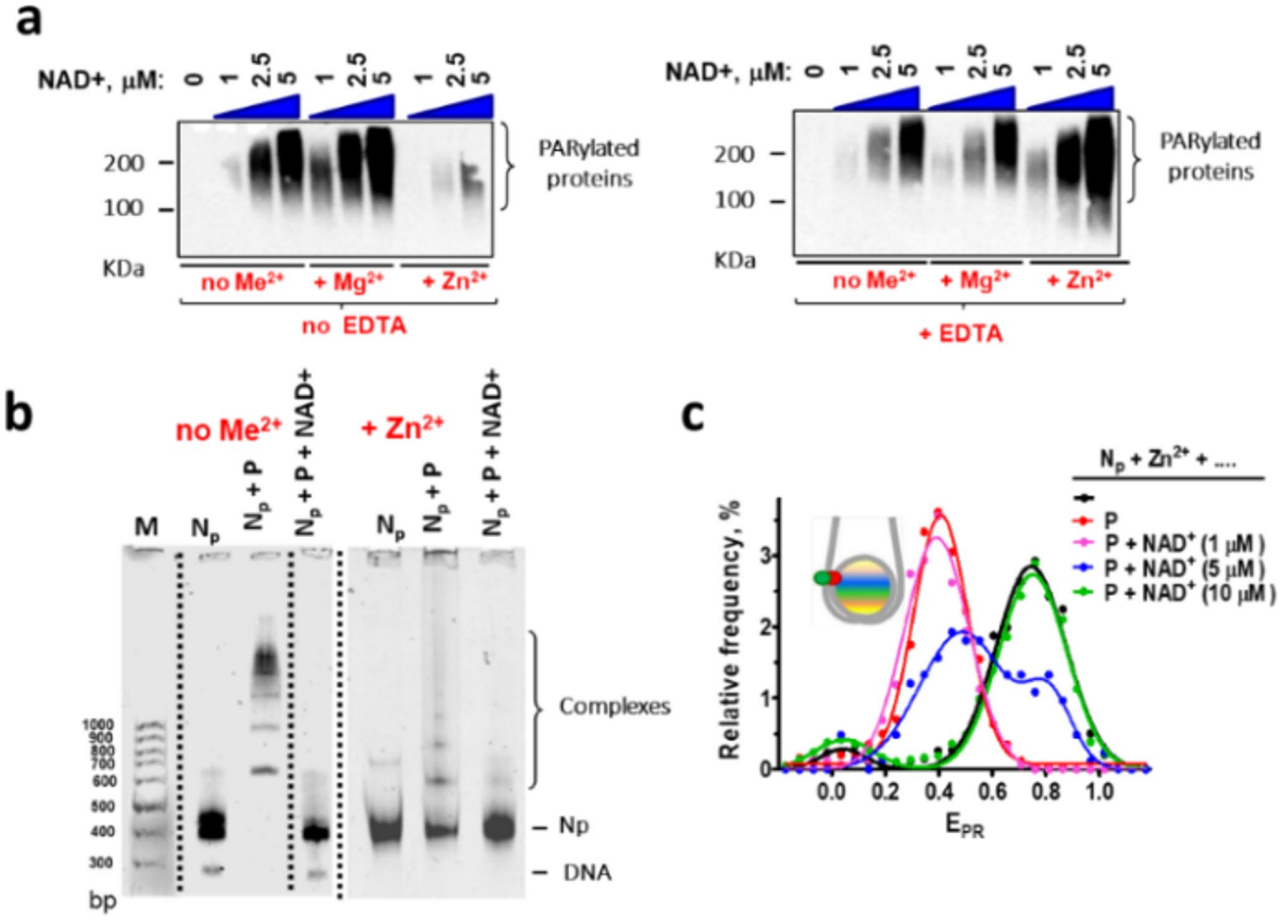
Enzymatic activity of PARP2 in the complexes with nucleosomes. **(a)** WB analysis of NAD^+^-dependent PARylation by PARP2 in PARP2-nucleosome complexes in the presence and absence of EDTA (10 mM), Mg^2+^ (5 mM) and Zn^2+^ (0.3 mM). Me^2+^: any divalent cations. Similar results were obtained in three independent experiments. Positions of protein size markers are indicated. **(b)** EMSA analysis of the complexes of N_p_ nucleosomes with PARP2 (100 nM) in the absence and presence of Zn^2+^ ions (0.3 mM) and NAD+ (10 µM). Me^2+^: any divalent cations. **(c)** Typical E_PR_ profiles of N_P_ nucleosomes and their complexes with PARP2 (P, 100 nM) after addition of different concentrations of NAD^+^. Samples were measured in the presence of 0.3 mM Zn^2+^ ions. Statistics: ~3000 particles per experiment

Addition of NAD^+^ substrate to the PARP2-nucleosome complexes activates a catalytic function of PARP2 and induces autoPARylation of PARP2 and PARylation of other proteins (Fig. 4a). The efficiency of PARylation by PARP2 depends on the concentration of NAD+ (Fig. 4a) and results in dissociation of PARP2 from the complexes with nucleosomes both in the presence and absence of Zn^2+^ ions (Fig. 4b-c).The dissociation of the complexes likely occurs because of electrostatic repulsion between negatively charged PARylated PARP2 and nucleosomal DNA [24, 57, 58]. The intact conformation of nucleosomes is restored after dissociation of PARP2 (Fig. 4b-c). Thus, Zn^2+^-dependent structural changes induced in nucleosomes by PARP2 are fully reversible.

The presence of Mg^2+^ or Zn^2+^ ions differentially affect the efficiency of PARP2-mediated PARylation: Mg^2+^ ions increase, while Zn^2+^ ions decrease the efficiency of PARylation (Fig. 4a). Addition of excess of EDTA, which chelates divalent ions, does not affect PARylation in the absence of divalent ions and fully reverses the effect of Mg^2+^ ions (Fig. 4a). Surprisingly, when Zn^2+^ ions are present in the buffer, addition of EDTA not only abolishes Zn^2+^-induced suppression of PARylation, but also strongly enhances the efficiency of PARylation (see Discussion).

## Discussion

PARP2 forms several complexes with a nucleosome without affecting the conformation of nucleosomal DNA (Fig. 1b, d, f, g): formation of the complexes occurs both in the absence of divalent cations and in the presence of Mg^2+^ or Ca^2+^ ions. A number of the complexes increase with the increase in concentration of PARP2: up to five complexes were detected by EMSA (Fig. 1b, f). At least three of the five complexes contain one nucleosome and likely contain different numbers of PARP2 molecules (Fig. 1c). Taking into consideration the ability of PARP2 to recognize DNA double-strand breaks, two PARP2 molecules might be bound to the ends of nucleosomal DNA. At least one PARP2 molecule might be bound to the nucleosome core region, due to the higher affinity of PARP2 to nucleosome as compared with DNA [28, 59]. The presence of the additional complexes could be explained by the ability of PARP2 to form homodimers at the concentration higher than 50–80 nM with the apparent dissociation constants of 152 and 101 nM in the absence and presence of DNA, respectively [4]. If this ability is preserved during the interaction with nucleosomes, formation of the additional PARP2-nucleosome complexes observed at 100–200 nM of PARP2 (Fig. 1b) could be attributed to dimerization of PARP2 molecules bound to the nucleosome and/or to the ends of nucleosomal DNA. It should be also noted that the size of PARP2 is relatively small, and several PARP2 molecules could potentially bind to a nucleosome without steric restrictions (Figure S7b).

Formation of PARP2-nucleosome complexes with different stoichiometry occurs nearly simultaneously (Fig. 1b) indicating similar affinities of the components of these complexes to each other. Indeed, this complicates both detection of these complexes and measurements of their dissociation constants (*K*_*d*_) by many techniques. These complications most likely explain the failure to recognize formation of different PARP2-nucleosome complexes in earlier experiments [28, 59], and relatively high values of *K*_*d*_, which were reported previously (76 nM [59] and 150 nM [28]). According to our measurements, 50% of nucleosomes are involved in the formation of complexes with PARP2 at 40 ± 10 nM PARP2 (Fig. 1b). Considerably lower *K*_*d*_ (11 nM) was reported for the PARP2 complex with the 18 bp duplex oligonucleotides [59], while the *K*_*d*_ values for the complexes of PARP2 with the 28 bp duplex oligonucleotides depended on the type of the introduced DNA defect and varied from 37 to 178 nM [2, 24, 25]. The *K*_*d*_ values of 11 and 37 nM probably describe the affinity of the 1:1 complexes, since direct binding of several PARP2 molecules to a short DNA fragment could be difficult due to possible steric interference between the bound PARP2 molecules, while dimerization of PARP2 occurs at much higher concentrations of PARP2 [4]. Affinity of PARP2 for longer DNA (147 bp) is considerably lower, with *K*_*d*_ values variable in the range from 840 nM for intact DNA to 58 nM for DNA having a gap [28, 59].

**Fig. 5:**
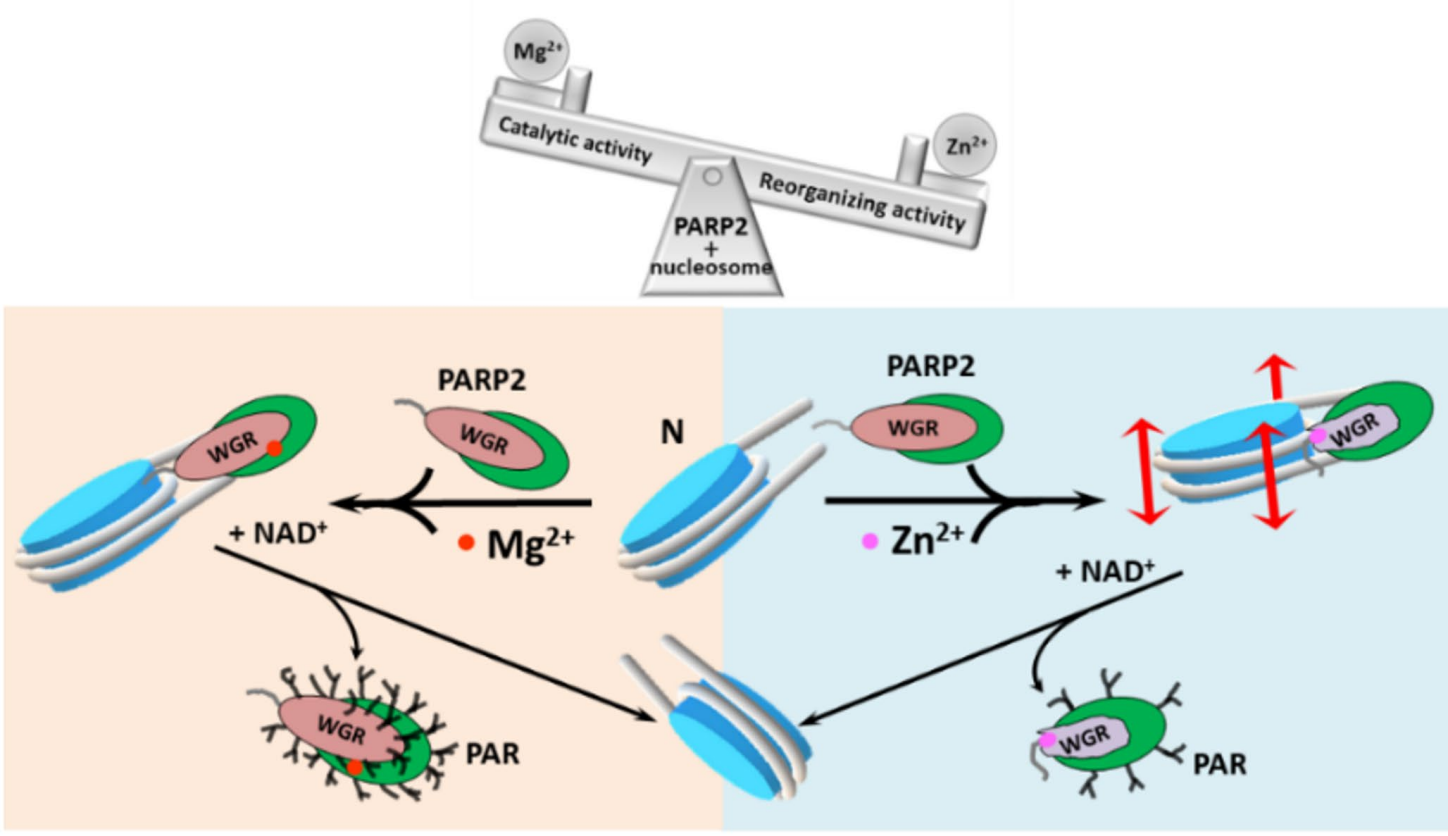
Functioning of PARP2 is differentially controlled by various divalent cations. Mg^2+^ and Zn^2+^ ions interact with the WGR domain of PARP2 and affect catalytic activity of PARP2, increasing or decreasing it, respectively. Additionally, Zn^2+^ ions mediate reorganization of the structure of nucleosomes by PARP2, while Mg^2+^ ions do not. NAD^+^-dependent catalytic activity of PARP2 results in autoPARylation of PARP2 and its dissociation from the complexes with nucleosomes; the released nucleosomes restore or preserve their native structure in the presence of Zn^2+^ or Mg^2+^ ions, respectively. Thus, transient alterations in the concentration of divalent cations in cells could regulate a balance between catalytic and nucleosome reorganizing activity of PARP2. N- nucleosome. Red and magenta circles are Mg^2+^ and Zn^2+^ ions, respectively. PAR – poly(ADP-ribose) chains

The presence of Zn^2+^ ions in solution results in the PARP2-mediated structural reorganization of nucleosomal DNA (Fig. 2). This effect is most likely mediated by direct interaction of Zn^2+^ ions with the WGR domain of PARP2, resulting in a local alteration in the secondary structure of at least the WGR domain of the enzyme and accompanying changes in nucleosome structure (Figs. 1h, 2 and 3). Binding of PARP2 to DNA occurs through the N-terminal region and WGR domain of the enzyme [2, 24, 25], consistent with the localization of ZnBS in the WGR domain. The structural changes induced in nucleosomes by PARP2 in the presence of Zn^2+^ ions are almost completely reversible after dissociation of the autoPARylated enzyme (Figs. 4c and 5).

Bioinformatic analysis predicted two probable ZnBSs in the WGR domain formed by residues E97, C98, H160 (site 1) and H106, C109, E138 (site 2) (Fig. 3a). ZincBind database [56] contains 44 structures of proteins having similar ZnBSs, including five human proteins. ZnBSs consisting of three amino acids are commonly found in enzymes and may have pico- and femtomolar affinity to Zn^2+^ ions [60, 61]. According to our measurements, the apparent dissociation constant of the complex between Zn^2+^ and the WGR domain of PARP2 is ca. 2 µM (Figure S4), but actually it can be smaller because residual EDTA that was present in the reaction solution definitely competed for the binding with Zn^2+^ ions. Other divalent cations, Mg^2+^ and Ca^2+^, also bind, with the lower affinity, to the WGR domain (Figure S5).

Interacting with PARP2, Mg^2+^ and Zn^2+^ ions affect catalytic activity of PARP2, increasing or decreasing it, respectively (Fig. 4a). To modulate the enzymatic activity of PARP2, the binding site for divalent cations should be localized at or very close to the catalytic domain of PARP2. However, we were unable to find any potential sites of Zn2 + binding within the catalytic domain using bioinformatics approaches and computational modeling. Since EDTA was able to cancel the effect of Mg^2+^ on PARP2 activity in the complex with the nucleosome (Fig. 4a), the binding site of Mg^2+^ is likely exposed to solution. EDTA was also able to change the effect of Zn^2+^ on the PARP2 activity from inhibiting to stimulating (Fig. 4a**)**, while it was not able to cancel the effect of Zn^2+^ on the structure of PARP2-nucleosome complex. The data confirm the existence of two sites of Zn^2+^ binding in PARP2; the sites are functionally different. One site is exposed to solution and affects the catalytic domain of PARP2. This site is responsible for the negative regulation of PARP2 activity with Zn^2+^. The second site of Zn^2+^ binding is responsible for the altered mode of PARP2 binding to the nucleosome. It is likely positioned in the WGR domain at the interface of PARP2-nucleosome interaction and is hidden inside the complex and therefore inaccessible to EDTA. This binding site is also responsible for the enhancement of PARP2 catalytic activity, when Zn^2+^ is removed from the first binding site by EDTA (Fig. 4a). This is consistent with the observation that the WGR domain makes a significant contribution to the DNA-dependent catalytic activity of PARP2 [62]. Note that Zn^2+^ ions specifically affect the autoPARylation activity, but not the PARP2 affinity for the nucleosome.

What is the Zn^2+^-mediated structural changes induced by PARP2 in a nucleosome? Three most obvious types of structural alterations could be proposed: DNA unwinding, nucleosome sliding and an increase in the distance between the gyres of nucleosomal DNA. The partial DNA dissociation (unwinding) from histones is initiated at the boundary of the nucleosome and could involve a variable number of base pairs. When DNA unwinding involves about 35 base pairs (as can be proposed considering a moderate decrease in E_PR_ between the labels localized at 35 and 112 bp), a decrease in E_PR_ between the labels localized at 13 and 91 bp or at 57 and 135 bp should be considerably higher than the decrease in E_PR_ between the labels at 35 and 112 bp. This difference was not observed in our experiments (Fig. 2a-c), indicating that unwinding of nucleosomal DNA is unlikely an explanation for the experimental data.

Nucleosome sliding is accompanied by simultaneous shift of both the donor and acceptor labels positioned on two supercoils of nucleosomal DNA in the same direction [63]; therefore, the E_PR_ values and the distance between labels will not be changed, in contrast to the results obtained in our studies. One possible exception is the sliding involving more than 13 bp, when the E_PR_ value for the labels located near the nucleosome boundary would decrease precipitously, while the E_PR_ values for the other two pairs of labels would remain unchanged; our data (Fig. 2a-c) do not support this mechanism. Therefore, a moderate decrease in E_PR_ occurring simultaneously between labels located on neighboring DNA supercoils near and far from the nucleosome boundary (Fig. 2a-c) is more likely explained by an increase in the distance between the DNA supercoils that occur along the entire nucleosomal DNA (Figs. 2d and 5).

The structure of full-length PARP2 and its complex with a nucleosome has not been determined yet, but a model structure of PARP2 predicted by AlphaFold2 algorithm [64] as well as a high-resolution structure of DNA-WGR domain complex (PDB entry 6F5B) are available. Using these models and our model of the WGR domain bound to zinc ions (Fig. 3a), a combined model of full-length PARP2 bound to DNA and zinc ions was constructed (Figure S7a). In this model the WGR, α-helical and catalytic domains of PARP2 form a compact structure; the N-terminal domain and the region connecting the WGR and α-helical domains are disordered. Potential ZnBSs are exposed to the solvent and are positioned relatively far from other PARP2 domains and from the interface between DNA and the WGR domain (Figure S7a). Note that the model does not account for the data on the shielding of ZnBS in the PARP2 complex with the nucleosome (Fig. 4c). However, the interaction of DNA with the N-terminal domain of PARP2, which is localized near one of proposed ZnBSs, and the mode of PARP2 binding to core nucleosome are not accurately described by this model.

Total concentration of zinc in cells is as low as 0.2–0.3 mM [65], and 30–40% of this zinc is localized in the nucleus [66]. Nuclear Zn^2+^ concentration increases during the S phase of a cell cycle compared to the G1 phase [67]. Some processes (for example, nitrosative stress) cause considerable changes in Zn^2+^ concentration in the nucleus [68] or, more often, in cytoplasm [69, 70]. In turn, changes in the concentration of cytoplasmic Zn^2+^ can induce quick response in the nuclear zinc concentration [71]. Consequently, PARP2 is either always associated with Zn^2+^ ions, or local short-term increases in the Zn^2+^ concentration within the nucleus can induce formation of the complexes of PARP2 with Zn^2+^. In both cases Zn^2+^ would change the mode of interaction of PARP2 with nucleosomes and the autoPARylation activity of the enzyme.

After the Zn^2+^-mediated changes in the interaction of PARP2 with nucleosomes, a decrease in the concentration of Zn^2+^ is not able to reverse these changes until autoPARylated PARP2 dissociates from the nucleosome (Fig. 3a), raising a question about the functional role of the Zn^2+^-mediated interactions of PARP2 with a nucleosome. The mechanism of DNA damage response (DDR) includes initial recognition of damaged sites by PARP1 and PARP2, activation of these enzymes in complexes with DNA, and autoPARylation and PARylation of neighboring proteins including histones. Newly synthesized PAR polymers recruit specialized DNA repair proteins to the damaged sites, concomitant with dissociation of autoPARylated PARP1 and PARP2 from DNA [72]. It remains unclear why the damaged sites retain the increased affinity for DNA repair proteins after dissociation of PARP1 and PARP2, if before the binding of these enzymes, the sites were not recognized by the repair proteins. One possibility is that rearrangement of the nucleosome structure by PARP1 [34] and by PARP2 in the presence of Zn^2+^ (Fig. 2a-c) induces formation of the binding sites for some of the repair proteins.

Zn^2+^ and Mg^2+^ ions differentially affect autoPARylation activity of PARP2 (Figs. 4a and 5). AutoPARylation activity of PARP2, in turn, determines the time of PARP2 association with a nucleosome (time of accumulation of a negative charge leading to PARP2 dissociation from a nucleosome), the rate of recruitment of the repair proteins and the dynamics of the early stages of DDR [2, 24, 72]. AutoPARylation activity of PARP1 also depends on the concentration of divalent cations [73]. Furthermore, certain combinations of divalent cations were reported to synergistically potentiate autoPARylation activity of PARP1 [73], suggesting that divalent cations are active players in DDR involving both PARP1 and PARP2, and that transient changes in the concentration of the cations could modulate PARP1- and PARP2-dependent DDR dynamics, structure and length of PAR-polymers as well as an extent of PARylation of nuclear proteins that undergo this post-translational modification. Magnesium and zinc deficiencies are known to be associated with various pathologies [74–76], raising the question of whether ion-dependent modulation of PARP1 and PARP2 activity occurs in these cases and contributes to development of the diseases.

## Conclusions

In summary, our data show that, surprisingly, PARP2 that does not have any specialized Zn^2+^-binding domains like zinc fingers is, in fact, Zn^2+^-dependent protein, and Zn^2+^ ions mediate PARP2 activity and its interaction with nucleosomes. AutoPARylation activity of PARP2 is strongly modulated by divalent cations (Fig. 5); the effect of Zn^2+^ ions is more complex as compared to the effect of Mg^2+^ ions. Zn^2+^ ions can change the mode of PARP2 interaction with the nucleosome that in turn could affect the chromatin-binding and DNA damage recognition functions of PARP2. PARP2-induced reorganization of nucleosomes could induce formation of the sites of binding for some DNA repair proteins and likely affects multiple processes in the nuclei, such as transcription and replication, where the temporary partial reorganization of nucleosomes is required to facilitate propagation of RNA polymerases and replication fork. Catalytic activity inhibition caused by PARP2-targeted drugs potentially may also be affected by Mg^2+^ and Zn^2+^ ions.

## Electronic supplementary material

Below is the link to the electronic supplementary material.

Supplementary Material 1

Supplementary Material 2

Supplementary Material 3

## Data Availability

A model of Zn^2+^ binding to the WGR domain was deposited at 10.5281/zenodo.12635801. The datasets analyzed during the current study are available from the corresponding author on reasonable request.
