# Supplementary material for: Poly(ADP-ribose)polymerase 2 is zinc-dependent enzyme and nucleosome reorganizer
Source: Cell Mol Life Sci. 2025 Jun 30;82(1):267. doi: 10.1007/s00018-025-05785-8 (PMC12209172; doi:10.1007/s00018-025-05785-8)
Supplement: Supplementary file 1 — Supplementary Material 1 [file 18_2025_5785_MOESM1_ESM.pdf]

POLY(ADP-RIBOSE)POLYMERASE 2 IS ZINC-DEPENDENT NUCLEOSOME REORGANIZER

Natalya Maluchenko, Alexandra Saulina, Olga Geraskina, Elena Kotova, Anna Korovina, Grigory Armeev, Mikhail Kirpichnikov, Alexey Feofanov, Vasily Studitsky

SUPPLEMENTARY MATERIALS

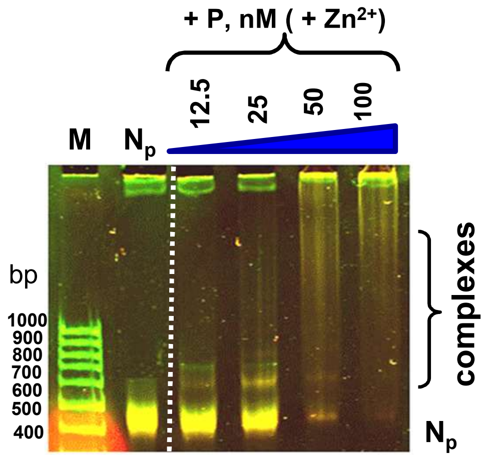

Figure S1. Analysis of Np nucleosomes and their complexes with PARP2 by non-denaturing PAGE. PARP2 (P) was added at concentration 12.5-100 nM in the presence of 0.3 mM Zn^{2+} ions. M – DNA markers. Aggregation of the complexes occurs at concentration of PARP2 above 50 nM and prevents their separation in the gel.

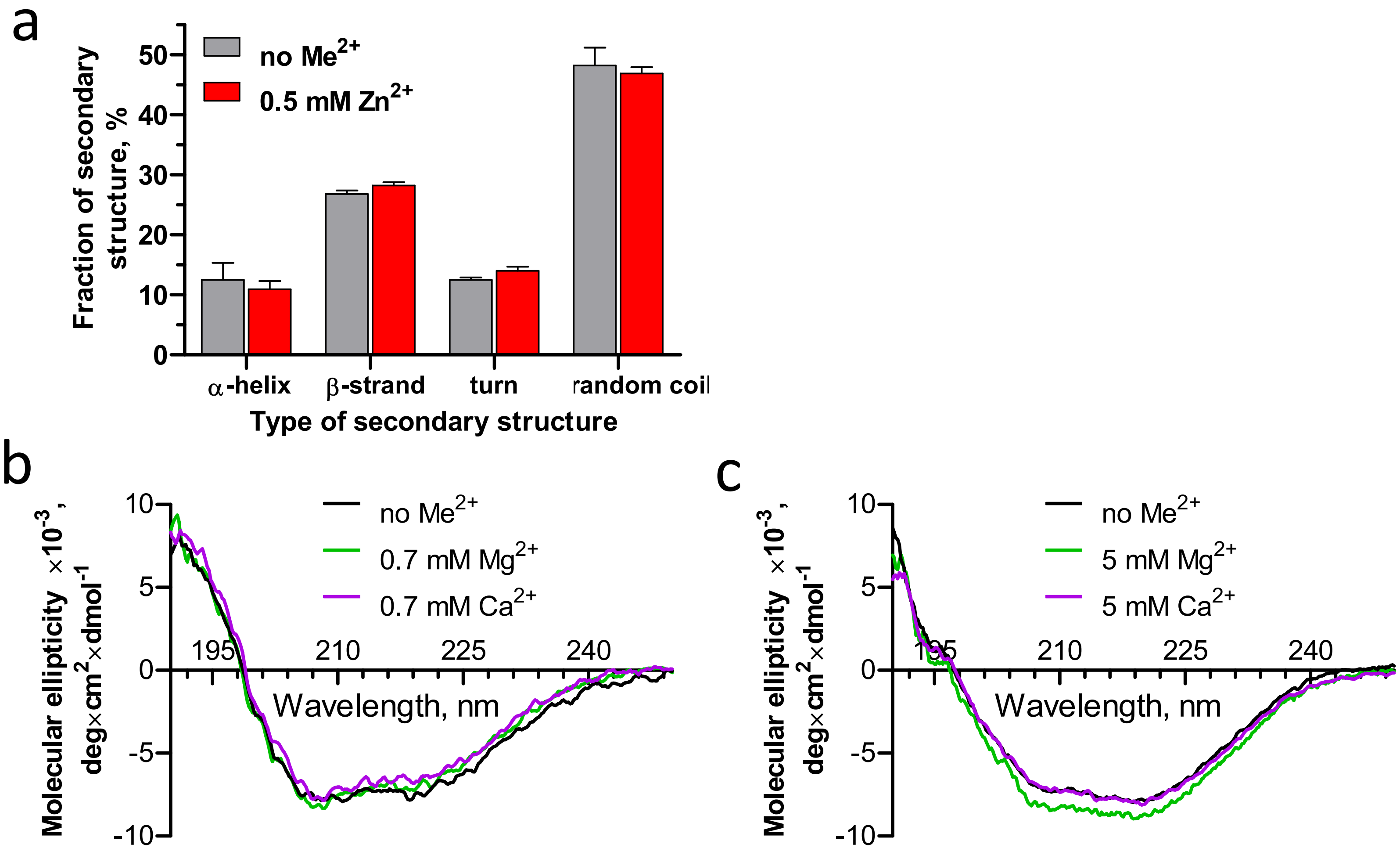

Figure S2. CD spectroscopy analysis of PARP2 (a, b) and its WGR domain (c). a) Changes in the content of the secondary structures in PARP2 induced by addition of Zn^{2+} ions (0.5 mM). Analysis of CD spectra presented in **Figure 2g**. **b,c)** CD spectra of PARP2 **(b)** and its WGR domain **(d)** in the absence or presence of Mg^{2+} or Ca^{2+} ions. Me^{2+} - divalent cations.

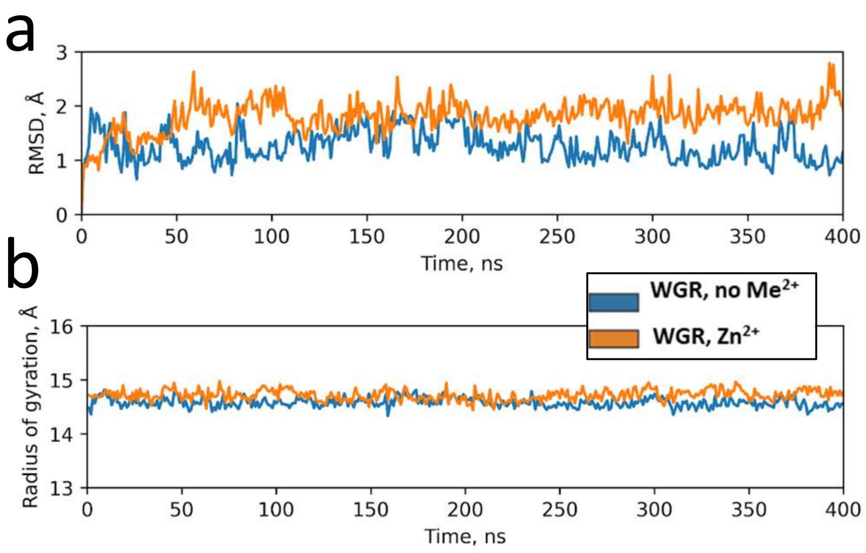

Figure S3. Analysis of the structure of the WGR domain of PARP2 in the presence and absence of Zn^{2+} **ions: MD simulations**. **a)** Changes in Root Mean Square Deviation (RMSD) of the Cα atoms of the WGR domain of PARP2 during the MD simulations. **b)** Changes in the radius of gyration of the PARP2 WGR domain during the MD simulations.

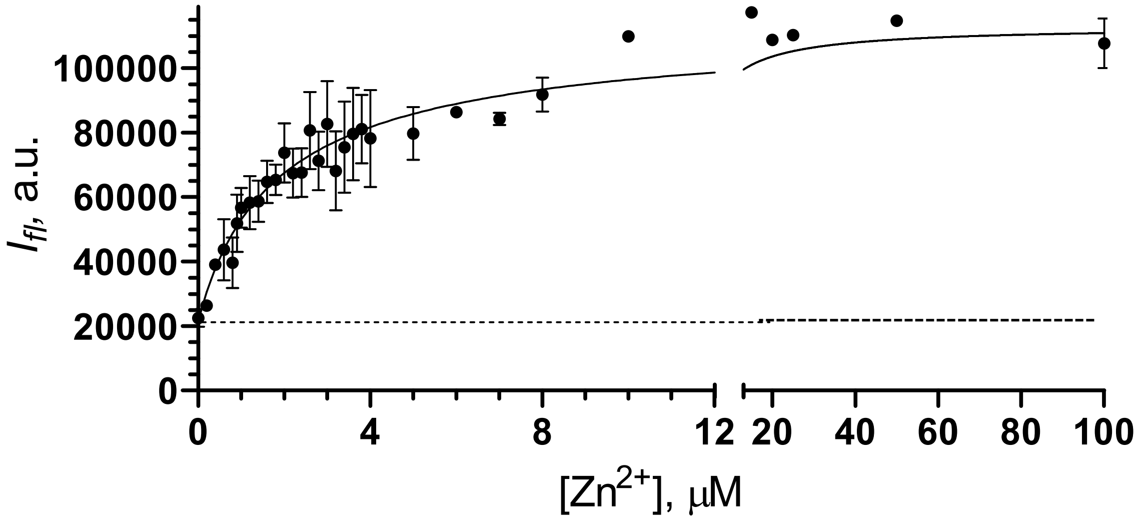

Figure S4. Changes in the intensity of fluorescence of tryptophane residues of the WGR domain as a function of Zn^{2+} ion concentration. Data were averaged over three independent measurements (mean ± SEM) and fitted with the equation describing the one site binding. Dashed line shows background fluorescence intensity in the absence of Zn^{2+} ions.

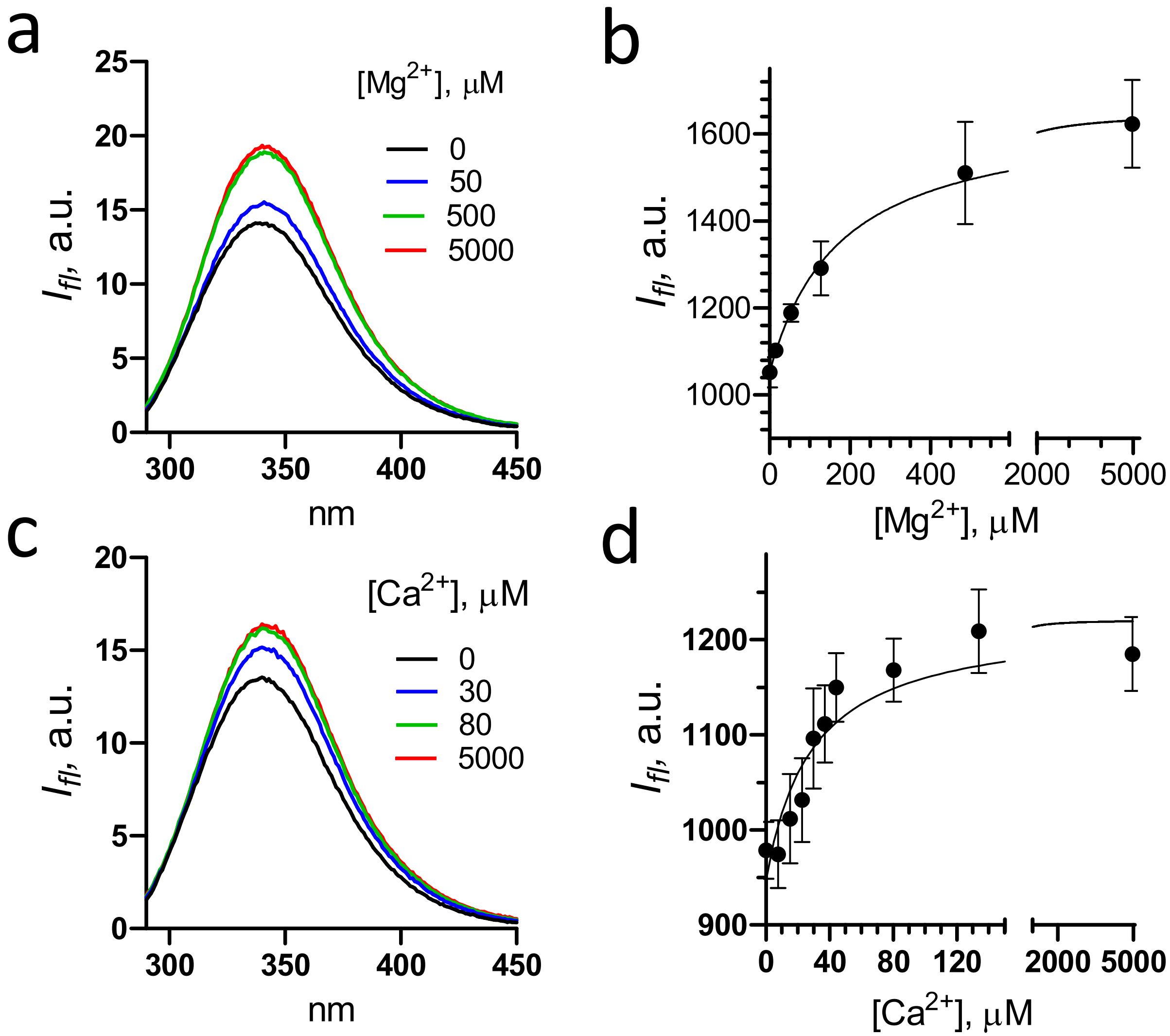

Figure S5. Tryptophan fluorescence of the WGR domain depends on concentration of Mg^{2+} and Ca^{2+} ions. a, c) Changes in the fluorescence spectra of the WGR domain upon addition of Mg^{2+} **(a)** and Ca^{2+} **(c)** ions. The measurements were conducted as in **Figure 3d**. **b, d)** Changes in the integral intensity of fluorescence of Trp residues of the WGR domain (5 μM) as a function of concentration of Mg^{2+} **(b)** and Ca^{2+} **(d)** ions. Data obtained in three independent experiments were averaged (mean ± SEM) and fitted with the equation describing the one site binding.

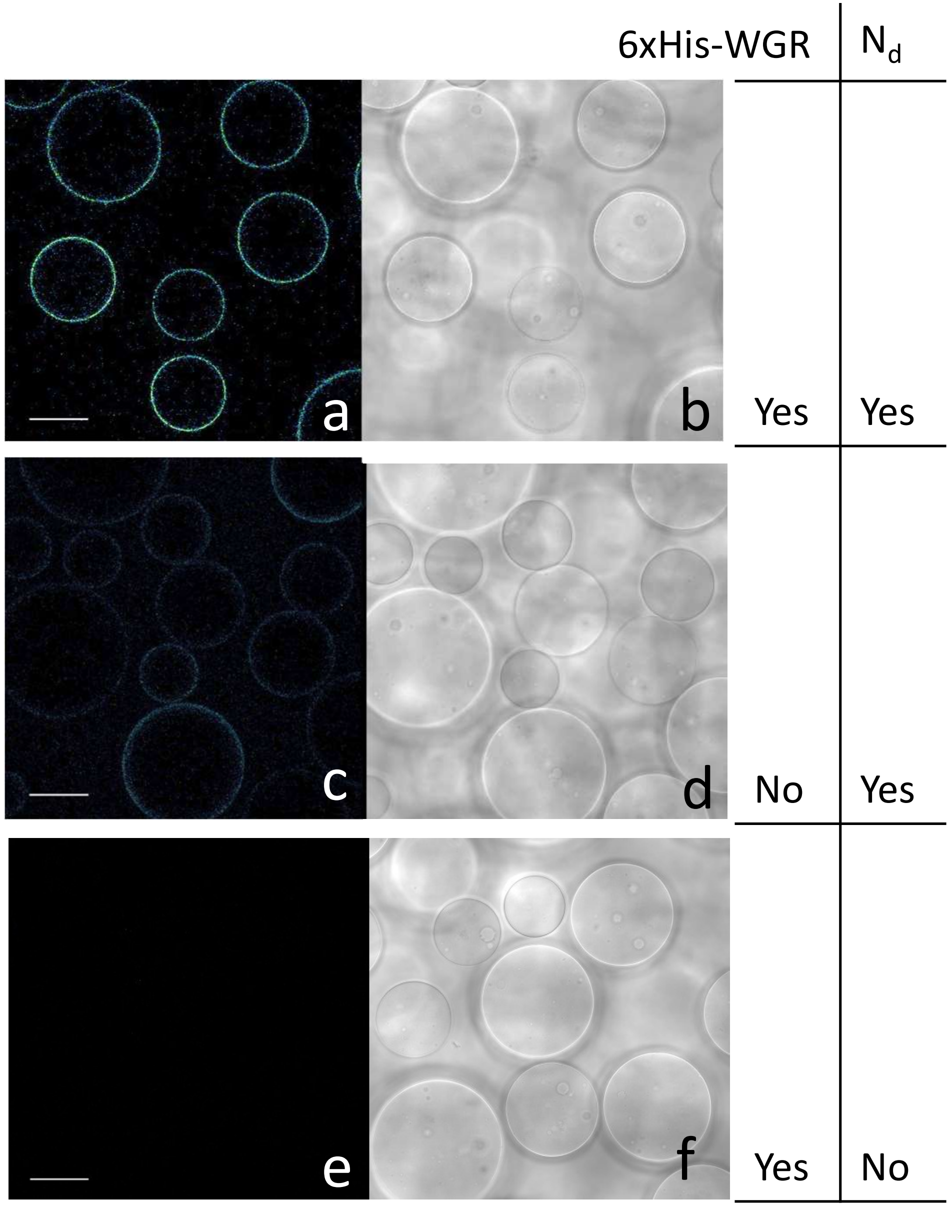

Figure S6. Confocal fluorescence (a, c, e) and transmitted-light (b, d, f) imaging of Ni-NTA beads with (a, b, e, f) or without (c, d) immobilized 6×His-WGR. The beads were additionally incubated with N_d nucleosomes **(a, b, c, d)**. The parameters of measurements and intensity scales in fluorescent images **(a, c, e)** are the same.

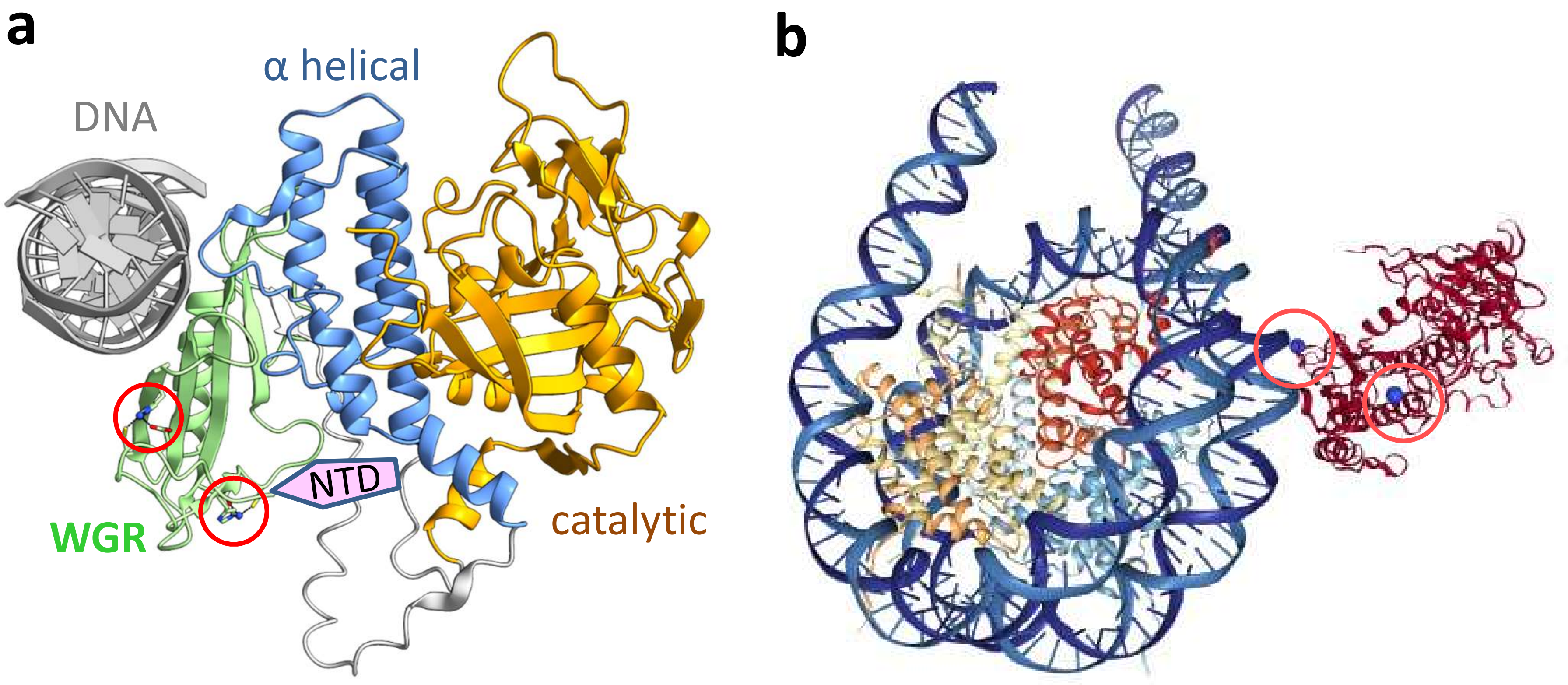

Figure S7. Models of complexes PARP2-DNA and PARP2-nucleosome. a) A model of DNA-bound PARP2 (without the N-terminal domain, NTD). The model was constructed using a structure of the complex of DNA with the WGR domain of PARP2 (PDB 6F5B), our model of the WGR domain in the complex with zinc ions (**Figure 3a**) and a model of PARP2 predicted by AlphaFold2 (AlphaFold2 AF_AFQ9UGN5F1). Red circles mark the proposed sites of Zn^{2+} binding. Pentagon shows the position of disordered NTD of PARP2. **b)** A model of PARP2 complex with a nucleosome. The model was obtained by inserting a fragment of DNA of the complex shown in **Figure S7a** into nucleosomal DNA. A site of PARP2 binding on a nucleosome was arbitrarily chosen. Red circles mark the proposed sites of Zn^{2+} binding. The exact modes of PARP2 binding to a nucleosome and DNA are likely to be different from each other (not shown). The size of PARP2 is relatively small, and several PARP2 molecules can potentially bind to a nucleosome without steric restrictions.
