# Supplementary material for: Poly(ADP-ribose)polymerase 2 is zinc-dependent enzyme and nucleosome reorganizer
Source: Cell Mol Life Sci. 2025 Jun 30;82(1):267. doi: 10.1007/s00018-025-05785-8 (PMC12209172; doi:10.1007/s00018-025-05785-8)

Fig. 1b

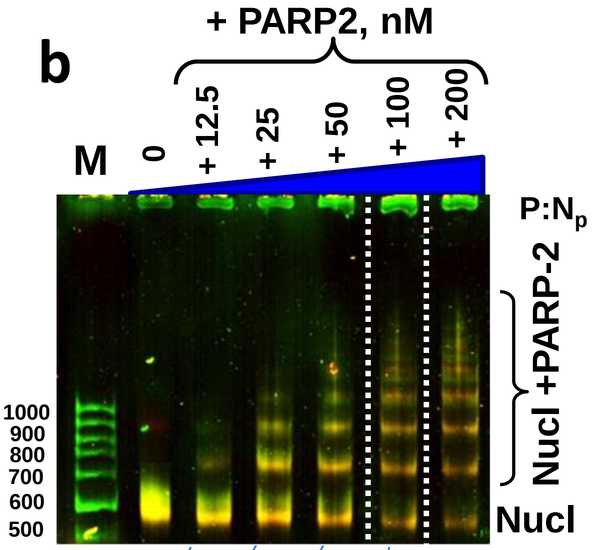

Fig. 1b RAW

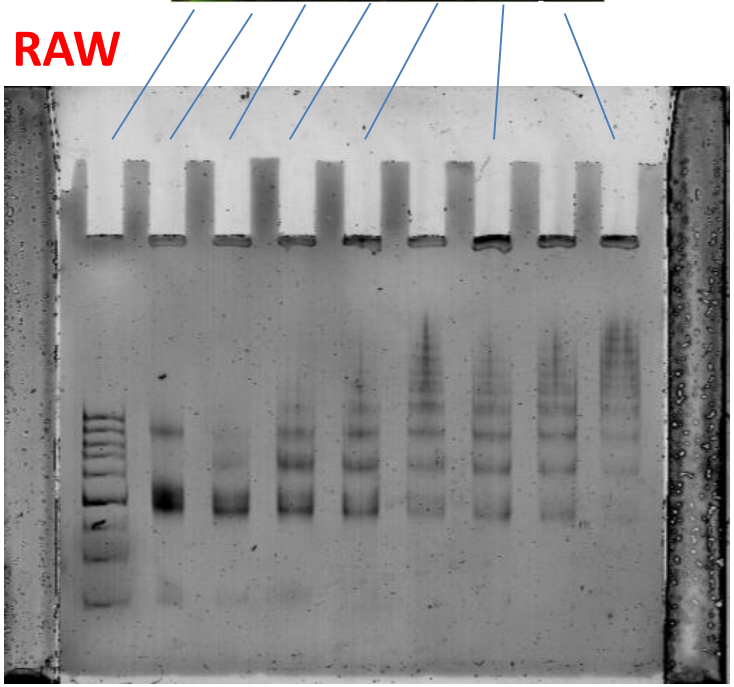

Gel in Cy3 channel

Fig. 1b RAW

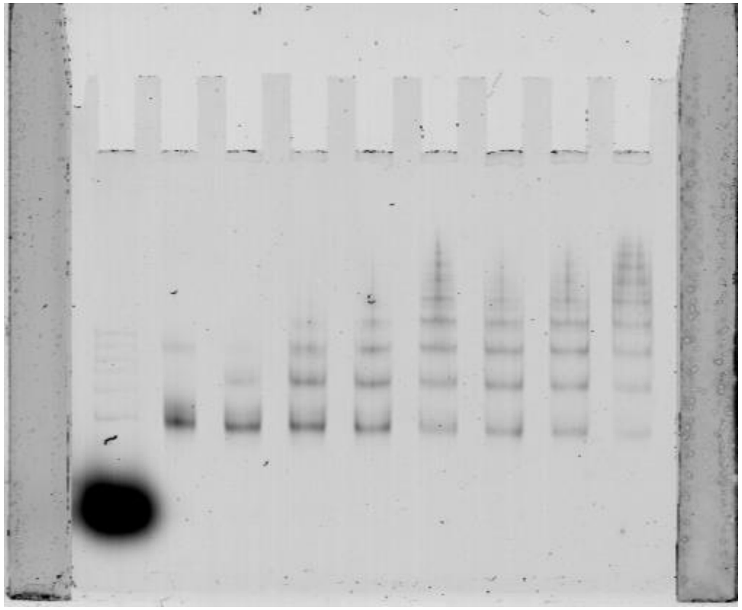

Gel in FRET channel

Fig. 1f

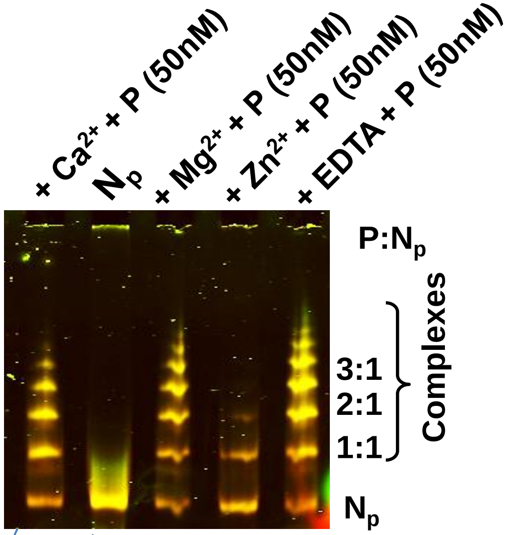

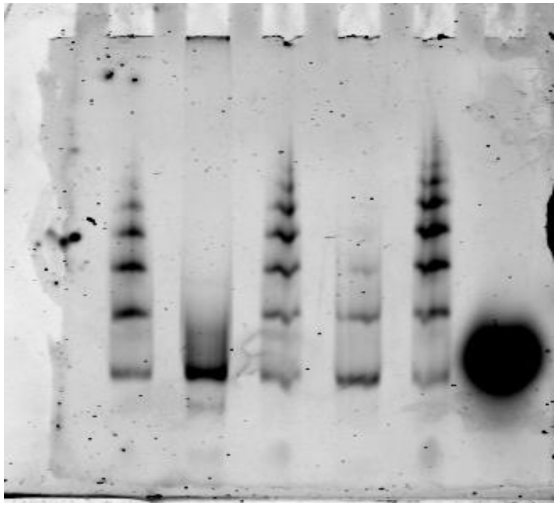

Fig. 1f RAW, channel Cy3

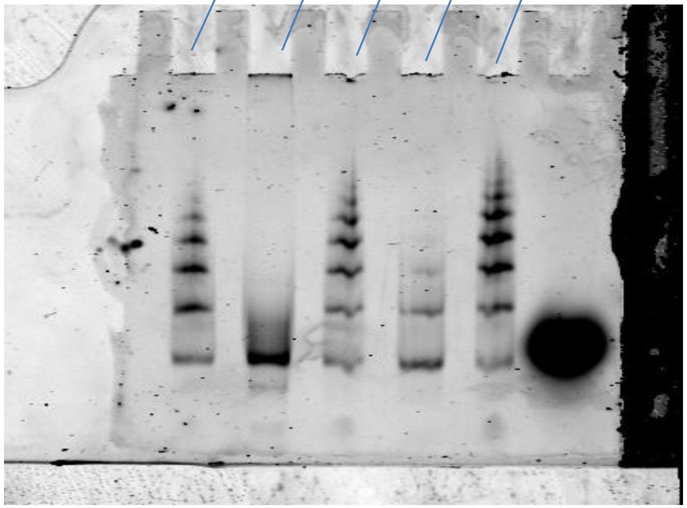

Fig. 1f RAW, channel FRET

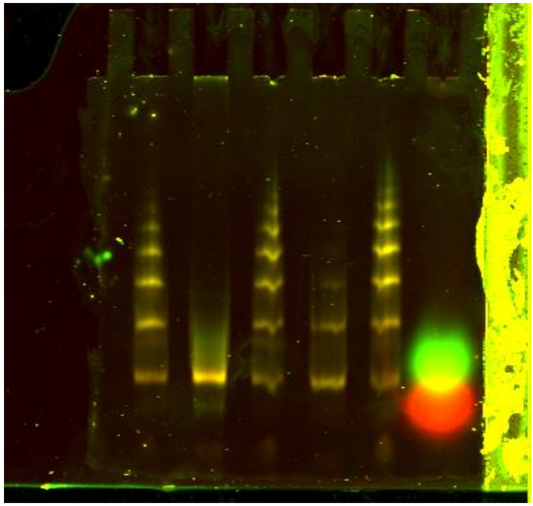

Fig. 1f RAW merge

Fig. 3f

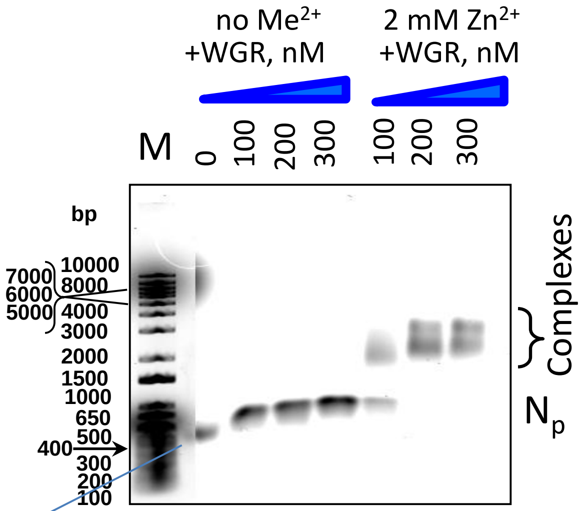

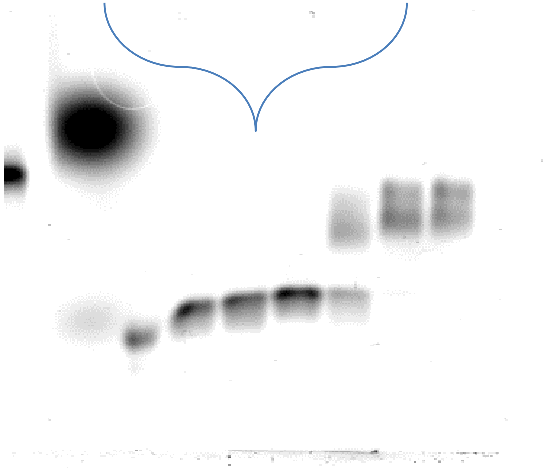

Fig. 3f RAW, channel Cy5

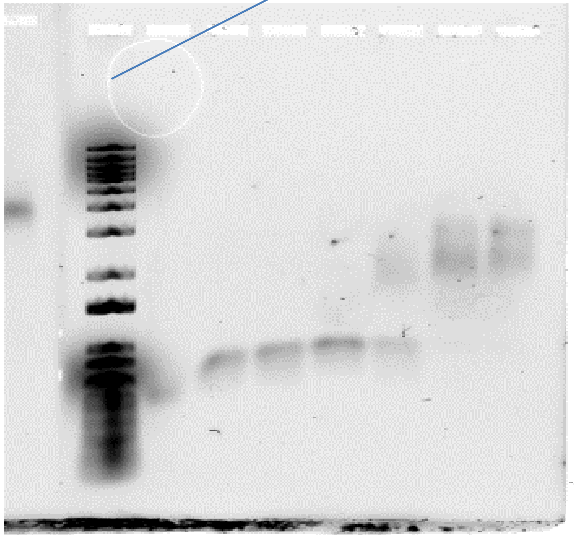

Fig. 3f RAW, channel Cy3

Fig. 4a

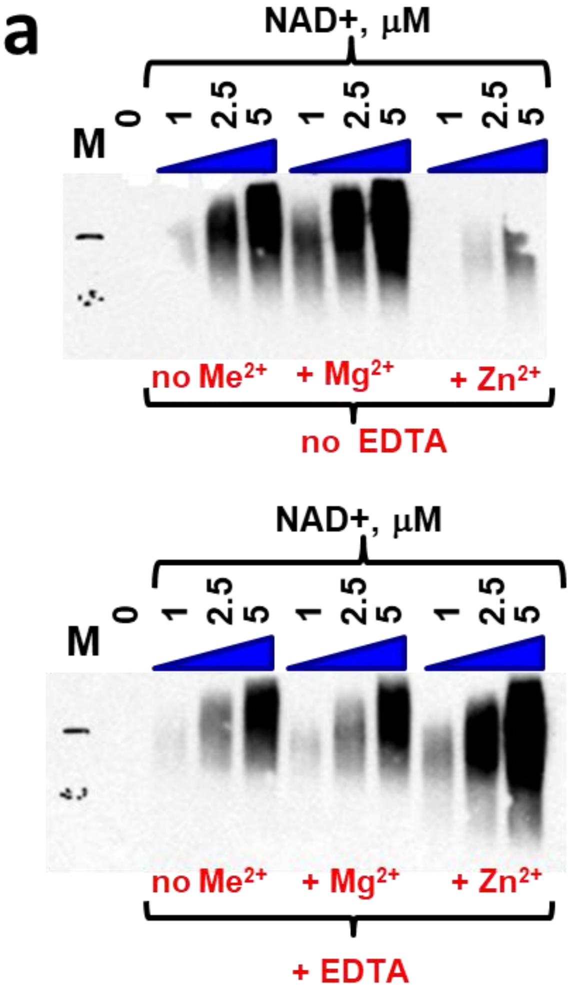

Fig. 4a RAW

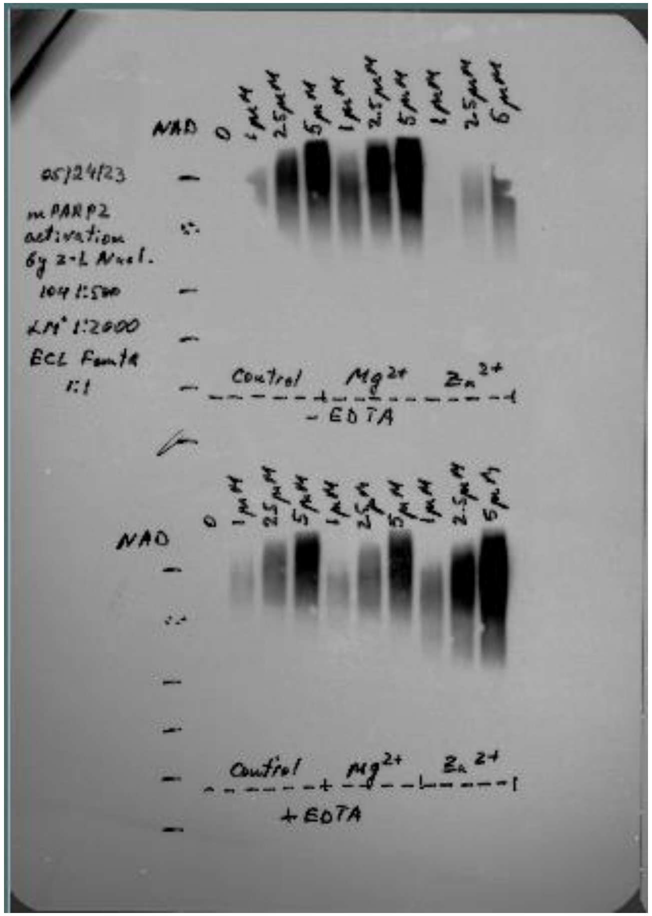

Fig. 4b

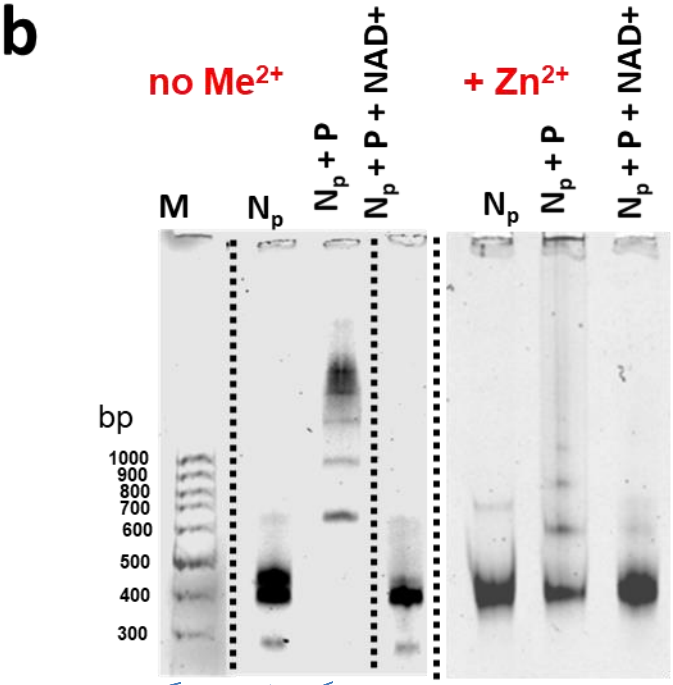

Fig. 4b RAW

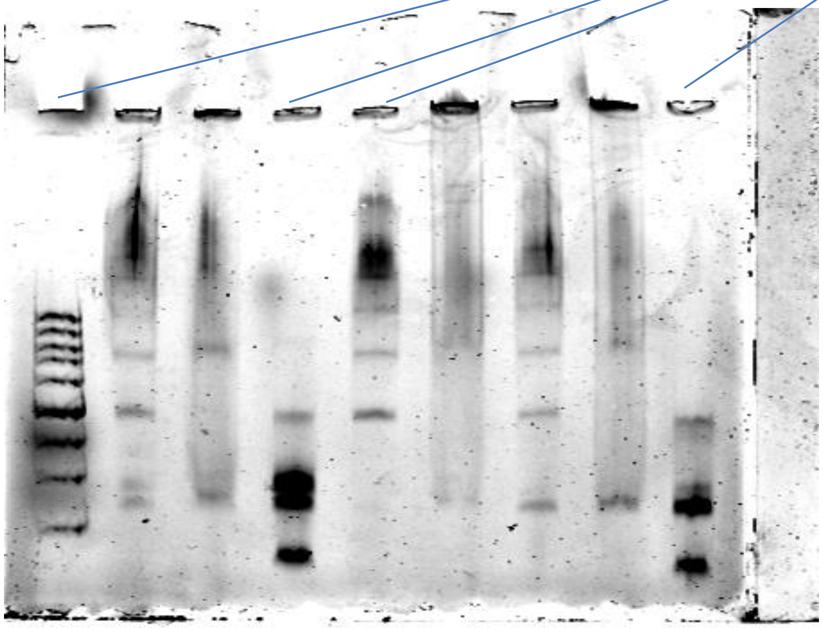

Fig. 4b RAW

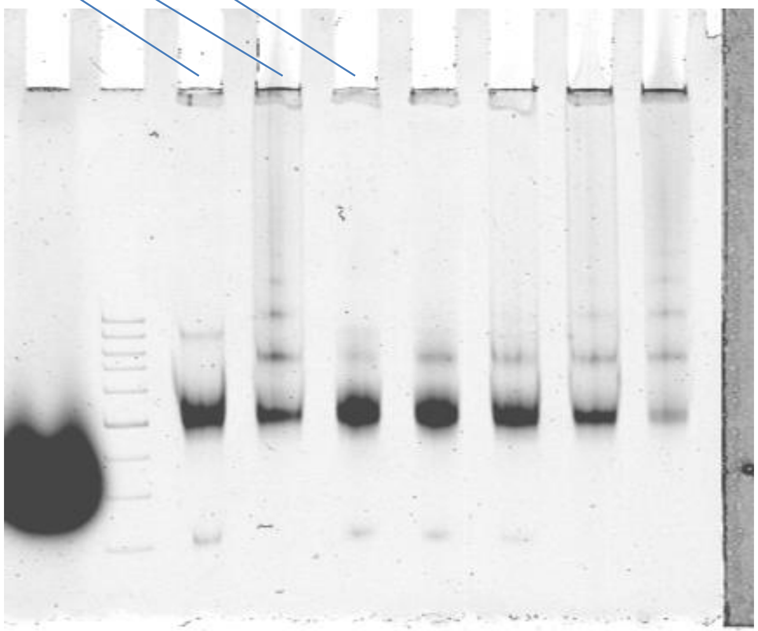

Supplement: Supplementary file 2 — Supplementary Material 2 [file 18_2025_5785_MOESM2_ESM.pdf]
